# Conformational Dynamics of hAgo2 Silencing: Decoding Functional Divergence across Human Argonaute Paralogs

**DOI:** 10.1021/acs.jcim.5c00194

**Published:** 2025-06-05

**Authors:** Antonella Paladino, Andrea Catte, Jorge Franco, Elisabetta Moroni, Silvia Rinaldi

**Affiliations:** † Institute of Biostructures and Bioimaging, IBB-CNR, Via Pietro Castellino 111, 80131 Napoli, Italy; ‡ Institute of Chemistry of OrganoMetallic Compounds, 201843 ICCOM-CNR, Via Madonna del Piano 10, 50019 Sesto Fiorentino, Firenze, Italy; § Institute of Chemical Sciences and Technologies, SCITEC−CNR, via Mario Bianco 9, 20131 Milano, Italy

## Abstract

RNA interference (RNAi) is a key mechanism for controlling gene expression, with Argonaute (Ago) proteins serving as core effectors of the RNA-induced silencing complex (RISC). By loading small noncoding RNAs, Agos target complementary messanger RNAs (mRNAs), leading to their direct catalytic cleavage or the activation of translational repression. Among the four human Ago isoforms (hAgo1–4), only hAgo2 exhibits catalytic activity, a feature not fully explained by structural differences alone. This study explores the structural and functional distinctions among hAgo isoforms, both in their unbound and bound states, using miRNA-20a as a model system. Microsecond-scale molecular dynamics (MD) simulations reveal insightful differences in structural flexibility and plasticity. Catalytically active hAgo2 demonstrates enhanced conformational dynamics, enabling essential structural transitions for efficient RNA silencing. Conversely, hAgo4 exhibits a more rigid conformation, consistent with its reduced catalytic activity. These findings suggest that human isoforms employ a conformational selection mechanism, where the interplay between structural rigidity and flexibility fine-tunes their functional roles. The isoform-specific dynamics unveiled in this study illuminate the functional specialization of human Ago isoforms, providing critical insights into their distinct role in RNA silencing. This understanding opens new possibilities for therapeutic innovation by modulating Ago-mediated pathways in an isoform-specific manner.

## Graphical abstract

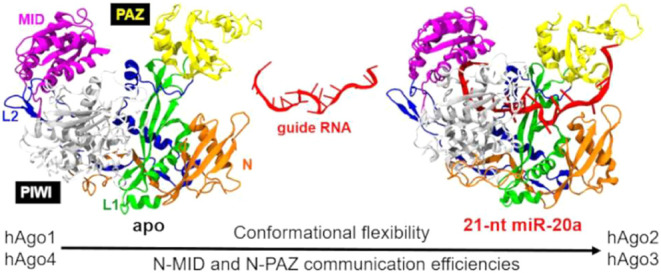

## Introduction

Cells adopt multiple strategies to control biochemical pathways. One particularly powerful mechanism relies on gene silencing mediated by regulatory RNA, known as RNA-interference (RNAi), which directly controls protein expression by targeting messanger RNA (mRNA). Central to this process are Argonaute (Ago) proteins, the main effectors of the complex assembly named RISC (RNA-induced silencing complex). When loaded with small RNA molecules (like micro RNAs, miRNAs, and small interfering RNAs, siRNAs), Ago proteins recognize complementary mRNA and either catalyze endoribonucleolytic cleavage of target RNAs or recruit factors for translational silencing and target destabilization. The entire functional cycle must lean on a precise and modular structural adaptation of RISC to perform all sequential steps that trigger RNA-silencing, including RNA loading, target recognition, cleavage, and recycling.^1–3^ Despite its biological significance, the intricate structural dynamics underlying these processes remains elusive, leaving critical aspects of RNA silencing poorly understood.

Recent years have witnessed extensive efforts to unravel various aspects of Argonaute-mediated gene regulation, encompassing the structural and biochemical features of the Ago proteins in their active and inactive states, the assembly of the RISC complex, the specificity of target recognition, and the catalytic mechanism of silencing. These studies have provided valuable insights, drawing parallels across the three domains of life.^1,3–10^

In humans, Ago proteins (hAgos) are critical for gene regulation, and their dysfunction has been linked to a range of diseases, including neurodegenerative disorders and cancers.^10–12^ Four human Argonaute isoforms have been identified (hAgo1–4), with hAgo1, hAgo2, and hAgo3 being the prevalent variants, whereas hAgo4 is only scarcely detectable.^1,13^ The differential expression of Ago paralogs across human cell lines add an additional layer of regulatory complexity, as miRNA function can be influenced by the specific hAgo protein engaged. Consequently, dysregulation of a specific paralog can lead to diverse effects on both physiological and pathological states. In this framework, a deeper understanding of the mechanisms governing hAgo proteins could lead to the development of novel therapeutic strategies targeting these pathways.

A crucial step in the RNA-silencing pathway is the establishment of an optimal miRNA-mRNA pairing within the hAgo RNA-binding complex. This process encompasses the loading of the miRNA in its double-stranded form, selection of the guiding miRNA strand, ejection of the unused (passenger) strand, target recognition and degradation (slicing), and the eventual release and recycling of the RISC complex.^3^ To accomplish these functions, hAgos must undergo a series of functional conformational transitions, triggered by interactions with several partners and favored by pronounced structural plasticity.^14–16^ Although a comprehensive understanding of these events in humans remains elusive, valuable insights have been derived from analogous steps characterized in *Drosophila melanogaster*.^3,6,32,33^ Crystal structures of Argonaute proteins from bacteria,^17–19^ archea,^20^ yeast,^21^ and humans^22–27^ reveal a conserved bilobed architecture, with one lobe comprising the N- terminal and the PAZ (PIWI-AGO-ZWILLE) domains, and the other lobe formed by the MID and PIWI (P element-induced wimpy testis) domains, connected through L1 and L2 linker domains (Figures 1 and S1). A central cleft formed between the two lobes accommodates the nucleic acid guide and its complementary RNA target, with the 3′ hydroxyl group and 5′ phosphate of the guide binding to the PAZ and PIWI domains, respectively. Structurally similar to ribonuclease H, the PIWI domain contains an RNase H-like tetrad responsible for target mRNA cleavage in catalytically competent Argonautes. Despite the highly conserved bilobed fold, human Agos have evolved additional structural elements, such as extended loops and secondary structures, that fine-tune and differentiate the protein functional dynamics, providing a foundation for exploring their unique structural and functional properties. The high degree of sequence conservation (>80%) among the paralogs results in a superposable multidomain architecture characterized by a clamp-like arrangement that binds RNA. Interestingly, most sequence variation is localized to the N-terminal domain whereas the MID and PIWI domains exhibit greater conservation (Table S1).^28,29^ While all four human Argonaute proteins are capable of binding short RNAs, only a subset displays catalytic activity, raising intriguing questions regarding their functional differences.^10,13,30^ Interestingly, sequence and structural variation alone fails to fully explain the biological differences reported so far (Figure 1), suggesting the existence of additional regulatory mechanisms.^27,31^ These may involve the availability and functional impact of interacting partners, alongside other less understood mechanisms that contribute to the precise modulation of Argonaute isoforms in response to specific cellular contexts and demands.

**Figure 1:**
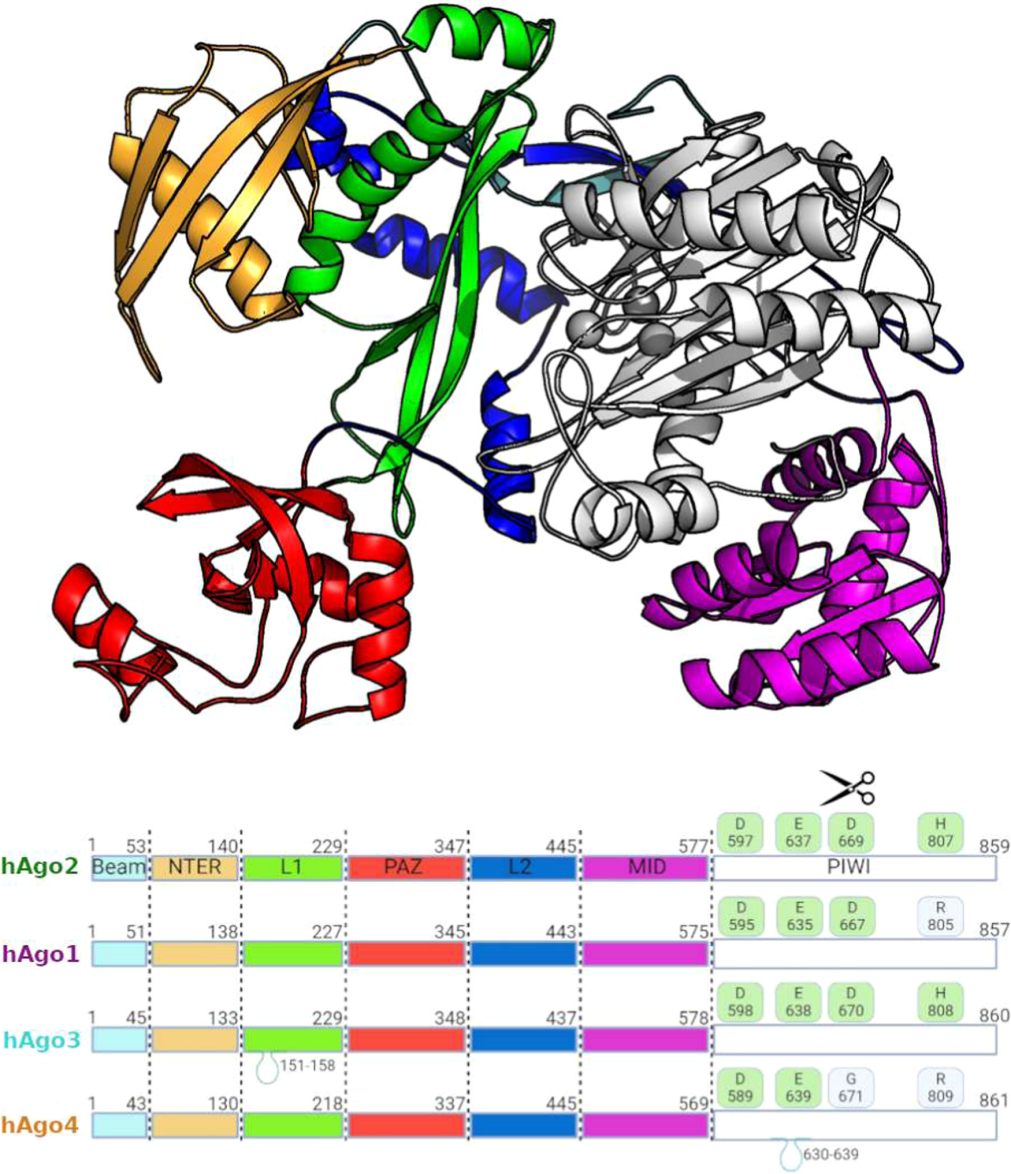
Representative Argonaute protein model. The X-ray crystal structure of hAgo2 (PDB ID:4Z4C) is rendered in cartoon representation and colored according to the domain classification given below. Gray spheres indicate residues of the catalytic site. A schematic alignment at the bottom is provided to highlight insertions and differences in the catalytic tetrads among the four isoforms.

For instance, among the four isoforms, only hAgo2 has been consistently reported to possess catalytic activity. Efficient endonucleolytic cleavage requires a fully conserved DEDH catalytic tetrad, which is intact in hAgo2 and hAgo3 but diverges into the inactive forms DEDR and DEGR in hAgo1 and hAgo4, respectively (Figure 1). Additionally, hAgo3 and hAgo4 lack two short sequence elements within the N-domain that are thought to be important for catalytic activity.^28,31^ Replacement of the hAgo3 N-domain with that of hAgo2 in chimeric constructs fully restored catalytic activity, underscoring the functional relevance of this region. In contrast, neither restoring the catalytic tetrad nor replacing the N-domain in hAgo1 was sufficient to rescue its catalytic activity. A similar outcome was observed for hAgo4, where correction of the catalytic tetrad failed to recover cleavage function, likely due to a short insertion in the PIWI domain that mispositions key residues and disrupts the active site. Notably, the deletion of this insertion had no apparent effect on its activity.^21,32^ Furthermore, hAgo1 and hAgo4 harbor additional repressive elements that may hinder the restoration of catalytic activity. For example, specific amino acids within the PIWI domain of hAgo1 (P670, P675, L674) impair slicing efficiency (Figure S1), by inducing a structural bent that interferes with the proper guide/target duplex positioning.^33,34^ Interestingly, hAgo3 has uniquely demonstrated the potential to adopt an active catalytic state under specific conditions,^27^ revealing a finely tuned, context-dependent regulatory mechanism driven by subtle factors that extend beyond sequence or structure alone. Collectively, these findings demonstrate that the presence of an intact active site motif is not sufficient to confer slicer activity. Instead, catalytic competence emerges as a multifaceted trait governed by a combination of structural, sequence-specific, dynamics and regulatory factors.^21,32^

To gain deeper insights into this critical issue, this study explores the structural and functional differences among the four human Argonaute isoforms, with a particular focus on the unique catalytic efficiency of hAgo2. Through extensive microsecond-scale molecular dynamics (MD) simulations, we dissect the sequence, structure, and dynamic features that drive functional specialization. Additionally, we examine the interaction of these proteins with a 21-*nt* miRNA-20a to uncover the determinants of binding specificity.

Our findings reveal distinct structural and dynamic behaviors that likely underpin hAgo2’s superior silencing efficiency, offering new mechanistic insights into its role as the primary slicer in RNA silencing pathways. By identifying isoform-specific features, our work contributes to a deeper understanding of Argonaute regulation, with potential implications for therapeutic strategies aimed at selectively targeting or modulating specific isoforms to enhance RNA-based interventions. While extensive literature supports the biological relevance of our observations, experimental validation remains essential to confirm this evidence. Overall, this study provides a novel framework for advancing research into RNA silencing and exploring the therapeutic potential of human Argonautes.

## Methods

### Molecular Dynamics Simulations

#### Starting X-ray Structures

PDB ID:5W6V, UniProt:Q9UL18­(hAgo1); PDB ID:4Z4C, UniProt:Q9UKV8­(hAgo2); PDB ID:5VM9, UniProt:Q9H9G7­(hAgo3); PDB ID:6OON, UniProt:Q9HCK5­(hAgo4).

For the apo systems, guide-target RNAs molecules were removed from the crystal structures, and missing residues and fragments were modeled using Maestro (Schrödinger Suite 2021–3).^35,36^ In the binary complexes (hAgo-guide-miRNAs), additional refinements were performed to complete the full-length RNAs (RNA (5′-UAAAGUGCUUAUAGUGCAGGU-3′)).

Metal ions (Mg^2+^) were removed from the RNA-binding pocket before performing MD simulations.

#### MD Simulation Settings

MD simulations were performed using Amber software package (version 2022),^37,38^ with the ff14SB force field and TIP3P^39,40^ water model. Each system was simulated in two independent replicas, collecting trajectories of 1 μs in an NPT ensemble at constant temperature (300 K) and pressure (1 atm). Electrostatic energies were computed using the particle mesh Ewald method,^41^ and a 10 Å cutoff was applied for Lennard-Jones interactions. All bonds involving hydrogen atoms were constrained using the SHAKE algorithm,^42^ following previous MD protocols.^14,15^

### Analysis Tools

#### MD-Based Routine Analyses

Root-mean-square deviation (RMSD), root-mean-square fluctuation (RMSF), and radius of gyration were calculated using GROMACS tools.^43^ RMSD was calculated on the Cα atoms of the entire protein and individual domains, RMSF was computed for the residues of the RNA-binding pocket common to all four isoforms, and the radius of gyration was evaluated for the full protein structures. To correlate conformational rearrangements with internal dynamics of each protein system, distance fluctuations analysis was performed to describe the dynamic coordination between any two residues. The distance fluctuation DF*_ij_* is defined as the time-average mean square fluctuation of the distance *r_ij_* between Cα atoms of residues *i* and *j*

$${\mathrm{DF}}_{\mathrm{ij}}=\left\langle {\left(r_{\mathrm{ij}}-\left\langle r_{\mathrm{ij}}\right\rangle \right)}^{2}\right\rangle$$

where brackets indicate the time-average over the trajectory. Low DF values indicate highly coordinated residues.^44^

#### Geometric Determinants and PCA Analysis

Structural descriptors were identified^14–16,45^ and computed as follows: Distances between centers of mass (COM) of (1) PAZ–PIWI, (2) PAZ-N, (3) PIWI-N, (4) PAZ-MID, (5) NPAZ-MIDPIWI, (6) PIWI-L2, (7) PAZ-helix7, (8) L1-L2; angles between (9) PAZ–PIWI, (10) PAZ-N, (11) PIWI-N, (12) PAZ-MID, (13) NPAZ-MIDPIWI. The time evolution of molecular determinants from each isoform simulation was mean-centered and scaled to unit variance prior to performing PCA on the combined data set. The resulting coefficients were then used to assess each determinant’s contribution to the overall variance. To identify the principal components (PCs) most effective in discriminating among the four isoforms, we excluded the first two PCs, to avoid dominance by major conformational shifts, and considered PC pairs explaining at least 15 % of the cumulative variance. These were ranked by their Calinski–Harabasz scores, and the determinants with the highest absolute coefficients on the top-ranking PC pair were selected as the key discriminative features.

#### Energy Decomposition Matrix (EDM)

EDM was computed for the hAgo representative structure obtained through cluster analysis of the 1 μs MD trajectories using AMBER Cpptraj.^46^ A hierarchical agglomerative clustering method was applied with an epsilon distance cutoff of 3.0 Å, using average-linkage algorithm to generate clusters.

EDM matrix^44^ yields the interaction energies (*E_ij_*) between residue pairs, comprising all the nonbonded inter-residue atomic energy components (van der Waals and electrostatic) calculated on the most representative conformation.

$$E_{\mathrm{ij}}={E_{\mathrm{ij}}}^{\mathrm{el}}+{E_{\mathrm{ij}}}^{\mathrm{vdW}}+{G_{\mathrm{ij}}}^{\mathrm{solv}}$$

The diagonalization of the EDM matrix allowed the extraction of the first eigenvector, which identifies the most significant inter-residues energy couplings.

#### Electrostatic Nuclei

The definition of stability hubs is provided applying a nonbonded energy cutoff on the EDM profile, described by the first eigenvector, associated with the first eigenvalue recapitulating most of the nonbonded energy. Only hAgos amino acids endowed with an energy *E_ij_* ≥ 0.005 kcal/mol were retained and intradomain interactions were calculated.

#### Intra- and Inter-domain Interaction Network

Hydrogen bond network was calculated for the four isoforms using VMD routine, and classified based on intra- and inter-domain interactions. Salt-bridges were computed using Amber Cpptraj. Only interactions persisting over at least 5% of the total simulation time were considered. Bonds mediated by equivalent side chains atoms were averaged.

#### Definition of the RNA-Binding Pocket

The RNA-binding pocket was defined by selecting all residues located within 6 Å of the bound RNA molecule, based on the X-ray crystal structure of hAgo2. Residues conserved across all isoforms were identified to define a minimal pocket, which served as a reference for subsequent comparative analyses.

#### Computation of Survival Probability of Water Molecules in Lakes

The analysis of water molecule dynamics was carried out to determine how long individual water molecules remain within a specific spatial region over time. This property, referred to as the survival probability *P*(τ), is defined as the fraction of molecules located in the region at an initial time *t* that are still present at a later time (*t* + τ). The decay of *P*(τ) reflects both the mobility of the molecules and the size of the spatial region considered.

The survival probability *P*(τ) was calculated using the following formula

$$P\left(\tau \right)=\frac{1}{T_{n}}\sum_{t}^{T_{n}}\frac{N\left(t,\tau \right)}{N\left(t\right)}$$

where *N*(*t*, τ) is the number of water molecules remaining in the region at time (*t* + τ), *N*(*t*) is the number of water molecules present in the selected region at time *t*, and *T*_*n*_ is the number of time steps contributing to *P*(τ).

For this analysis, the calculations were performed using time windows ranging from τ = 0.1 to 1 ns. MD meta-trajectories were divided into intervals of 20 ns, excluding the first 100 ns of each trajectory to ensure the system reached equilibrium. The resulting final survival probability values *P*(τ) were obtained averaging across all time windows.

The computations were carried out using the water dynamics analysis tools implemented in the MDAnalysis software suite.^47–49^

To evaluate the water dynamics within the LAKE cavities for the four Argonaute isoforms, atomic selections were defined to capture water molecules residing in this region during MD simulations. Water molecules were considered to reside inside LAKE1 and LAKE2 if their oxygen atom was located within 4 Å of a specified group of atoms.

For each isoform, the residues used to define LAKE1 are as follows:

**hAgo1**: the side-chain atoms of residues 574, 621, 623, 854 and the backbone oxygen of residues 571 620, 785, 787, 769, 788, 791, 793, **hAgo2**: the side-chain atoms of 576, 623, 625, 856 and the backbone oxygen of 573, 622, 787, 789, 790, 791, 793, 795; **hAgo3**: the side-chain atoms of 577, 625, 627, 857, and the backbone oxygen of 574, 623, 788, 790, 791, 792, 794, 796; **hAgo4**: the side-chain atoms of 568, 615, 617, 858, and the backbone oxygen of 565, 614, 789, 791, 793, 795, 797.

For each isoform, the residues used to define LAKE2 are as follows:

**hAgo1**: the side-chain atoms of 713, 715, 721, 726, 728, 757, 760, the Cβ atom of 370 and the backbone atoms of 712; **hAgo2**: the side-chain atoms of 715, 717, 723, 728, 730, 759, 762, the Cβ atom of residue 372 and the backbone atoms of 714; **hAgo3**: the side-chain atoms of 716, 718, 724, 729, 732, 760, 763, the Cβ atom of residue 373 and the backbone atoms of 715; **hAgo4**: the side-chain atoms of 717, 719, 725, 730, 732, 761, and 764, the Cβ atom of residue 362 and the backbone atoms of 716.

#### Allosteric Communication Pathways

Allosteric communication pathways of each simulated system were determined using the representative protein structures from the top 3 clusters generated by the clustering analysis of MD simulations. These structures were transformed into networks of nodes, whose topology depended on the Kirchhoff matrix of inter-residue contacts, generated using the coordinates of α-carbons, and the edges were weighted with commute times between connected nodes. The Kirchhoff matrix of each analyzed structure was produced by a Gaussian Network Model (GNM) calculation performed with ProDy.^50–52^ The Markov model of network communication was applied to calculate hit and commute time matrices. The commute times matrices were employed to extract the shortest communication pathways of connected graphs of each representative structure using two MATLAB scripts, provided by Prof. Guang Hu and employed as described in his tutorial,^53^ and the Dijkstra’s algorithm^54^ implemented in the Matlab function *shortestpath*. For each shortest communication pathway, the commute time was calculated using an in-house Matlab script. Finally, python scripts were used to extract the top 15 shortest communication pathways containing the top 15 conserved residues for each cluster.

## Results

### Conformational Dynamics of hAgo Proteins

The overall domain organization of human Argonaute proteins (hAgo1–4) is highly flexible in the RNA-free state. This flexibility leads to a “breathing” motion of the bilobed architecture, shaping the RNA-binding channel. Hence, the proteins can adopt more open conformations, facilitating the accommodation of small RNA duplexes (Figure 1).^14,16,55^ Such high variability in the apo state may explain why all available full-length crystallographic structures of hAgo isoforms are resolved in RNA-bound forms.

Investigating the conformational dynamics of these proteins in their apo state is thus essential to uncover potential functional differences and for identifying distinct molecular recognition mechanisms underlying RNA binding. To this end, we employed a comparative computational approach based on microsecond-scale all-atom MD simulations, complemented by physicochemical and structural *ad hoc* analyses to study both global and local conformational changes and identify isoform-specific features.

Starting from the full-length crystal structures reported for the different hAgo isoforms (PDB IDs: 5W6V (hAgo1),^25^ 4Z4C (hAgo2),^7^ 5VM9 (hAgo3),^27^ 6OON (hAgo4)^26^) and removing the nucleic acid (as detailed in the Methods section), we run multiple replicas of unbiased MD simulations of the four isomers in their apo states for a 1 μs meta-trajectory *per* system.

RMSDs of the full-length backbone atoms indicate significant rearrangements in the initial frames of the simulation time, likely reflecting conformational relaxation following RNA removal (see Figure S2). Domain-specific RMSD averages show minor differences among isoforms, with larger variability observed for the PAZ domain (Table S2A). Interestingly, while hAgo1,3,4 exhibit comparable RMSD profiles, hAgo2 displays markedly higher dynamics in this domain. Previous studies have demonstrated that PAZ is the only domain responsible for capturing the guide miRNA at the 3′ end,^4^ linking its increased intrinsic flexibility to a distinct functional role of hAgo2 in recognizing miRNAs.

Moreover, although the overall size of the bilobed proteins is similar across all paralogs, with the radius of gyration (*R*_g_) stabilizing around 31 Å (Table S2B), the degree of interdomain opening between MID-PAZ and PIWI-N is more pronounced in hAgo2 and, to a lesser extent, hAgo3. This motion is driven by the larger flexibility of the PAZ domain and resembles the characteristic interlobe “breathing” motion, previously described (Figure S3).^15,16,55^

To identify structural signatures associated with the main opening/closing movement, we defined multiple geometric descriptors across the protein. Specifically, we selected combinations of distances and angles reported in the literature as important parameters for capturing these conformational changes. The goal was to establish correlations between macroscopic ’breathing’ motions of the bilobed structure and local conformational shifts, under the hypothesis that these local changes may either drive or respond to the overall structural dynamics. To identify the key factors guiding the distinct dynamic behaviors of the isoforms, we performed a Principal Component Analysis (PCA) on the time evolution of 13 structural descriptors (see Supporting information (SI) and Methods). The analysis focused on the population distribution in the essential subspaces defined by the resulting eigenvectors. The first two components, accounting for 55.4% of the total variance, were excluded to prevent the dominant bilobe opening motion from masking more subtle local rearrangements (see Figure S3). Instead, we selected the component pair PC3–PC5, which explains 19.2% of the variance and was ranked highest by the Calinski–Harabasz index for its ability to discriminate among isoforms (see Figure 2 and Methods). Inspection of the PCA coefficients highlighted that the distances between the center of mass (COM) of PAZ–PIWI and PIWI-L2 domains were effective discriminants of local conformational differences among the isoforms (see Figure 2). Here, hAgo4, and to a lesser extent hAgo1, display narrower distributions, indicating that the PAZ–PIWI and PIWI-L2 relative positioning undergoes smaller fluctuations during the simulation. In contrast, hAgo2 exhibits the greatest dispersion, with each isoform populating a broad conformational basin characterized by a wide range of PIWI–PAZ (approximately 15 Å) and PIWI-L2 (about 5 Å) distances. In addition to this shared conformational basin, hAgo2 and hAgo3 exhibit distinct, less populated conformational ensembles only accessible to their respective isoforms. These ensembles are characterized by shorter distances between the PIWI–PAZ and PIWI-L2 domains, indicating a stabilization of more compact bilobed structures.

**Figure 2:**
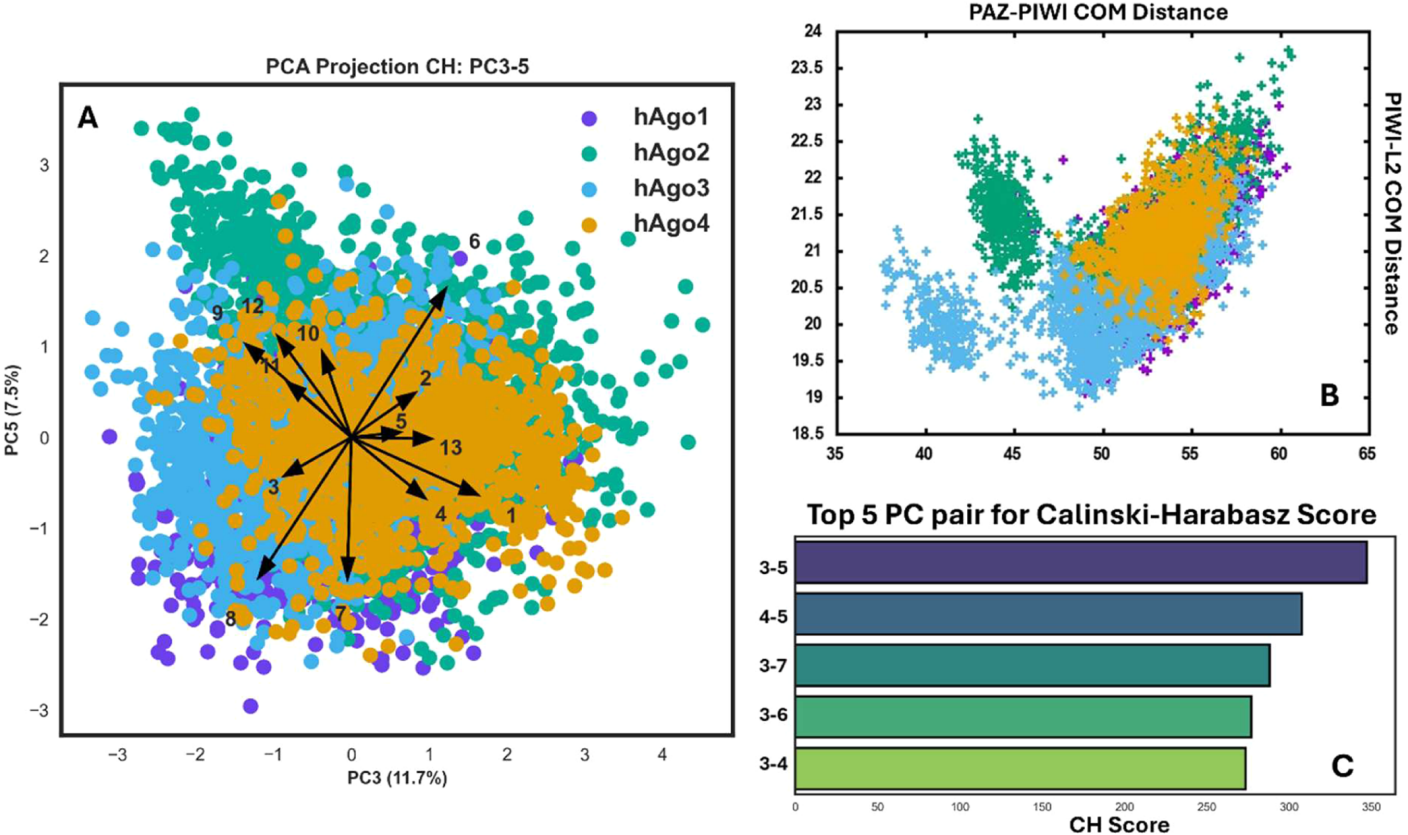
PCA analysis and Ensemble distribution. Principal Component Analysis (PCA) of structural determinants across four protein isoforms. (A) Projections of the structural determinants for each isoform are shown within the common essential subspace defined by Principal Components 3 and 5 and vectors representing the coefficients (i.e., the weights) of each structural descriptor are plotted in the same essential subspace, highlighting their relative contributions to the variance observed in the data set. (B) Distribution of values calculated as the center of mass (COM) distance between the PAZ and PIWI domains (*x*-axis) and between PIWI-L2 (*y*-axis) in the apo states of the four hAgo isoforms. (C) Top 5 Principal Component Pairs ranked by Calinski–Harabasz Score.

#### Structural Stability of hAgo Proteins

We next examined the energetics associated with the conformational changes driven by domain interactions and motions. The underlying hypothesis is that different combinations of interdomain interactions stabilize hAgo conformations associated with the isoform-specific functionalities. The average values of nonbonded interaction energies (electrostatic and van der Waals) between domains are shown in Table S3. As expected, these interactions primarily involve domains within the two lobes, as the opening motion between the lobes enclosing the RNA binding channel exceeds the 10 Å cutoff used to compute the interaction energies. Once again hAgo2 stands out as a distinct system, where a greater plasticity is reflected by lower interaction energies between the N and PAZ domains as well as the PIWI and MID domains.

Interestingly, hAgo4, the only isoform with no reported catalytic activity, shows a distinct behavior characterized by an asymmetric distribution of interaction energies, with stronger interactions between the N and PAZ domains, and weaker interactions between the MID and PIWI domains compared to hAgo3 and hAgo1, which display intermediate properties.

We then used the energy decomposition (EDM) method^44,56,57^ to map pairwise nonbonded interaction energies onto the 3D structure of Argonaute proteins. EDM is a valuable tool to evidence variable energetic regions across the proteins and specifically distinguish plastic/low-interacting vs rigid/high-interacting motifs. Analysis of the most representative structure of the four proteins (cluster #1 from hierarchical clustering, see Methods) shows significant differences among the domains in the EDM profiles, while highlighting the stabilizing role of the PIWI domain in all isoforms. hAgo1 and hAgo4 exhibit a more similar trend compared to hAgo2 and hAgo3 (Figure 3). Specifically, in hAgo1 and hAgo4, additional regions contribute to energetic stabilization alongside the primary role of the PIWI domain. Notably, the linker domains L1 and L2, connecting PAZ to the N and MID domains, respectively, emerge as significant contributors. The N domain also serves as a secondary hotspot for stabilization in hAgo1 and hAgo4, as evidenced by the number of intradomain hydrogen bonds formed during the trajectory. Compared to hAgo4, endowed with a higher number of stabilizing hydrogen bonds in the N and PIWI domains (see Table 1, Figure S4), the reduced domain stabilization caused by fewer hydrogen bonds present in hAgo2–3, aligns with the enhanced plasticity and flexibility required for functional states. This behavior reminds the mechanistic strategy described for thermophilic prokaryotic Argonautes (pAgos), where optimal activity at high temperatures is assured by loosely packed and partially disrupted dynamic structures.^58^ Overall, while the presence of secondary stabilization hotspots in hAgo1 and hAgo4 reallocates the central role of PIWI, leading to a more distributed pattern of energetic hotspots across the protein, hAgo2 and hAgo3 demonstrate a more polarized stabilization profile, dominated by the PIWI domain, reflecting distinct structural adaptation linked to their functional specialization (Figure 3).

**Table 1:** Intradomain Hydrogen Bonds in hAgo apo Conformations^a^

	**Nter**	**L1**	**PAZ**	**L2**	**MID**	**PIWI**
**hAgo1**	43 ± 5	31 ± 4	49 ± 5	32 ± 4	60 ± 5	129 ± 8
**hAgo2**	38 ± 4	35 ± 4	49 ± 5	34 ± 4	63 ± 5	136 ± 8
**hAgo3**	42 ± 4	36 ± 4	51 ± 5	33 ± 4	60 ± 5	133 ± 8
**hAgo4**	48 ± 5	33 ± 4	47 ± 5	32 ± 4	58 ± 5	142 ± 10

a Mean values and standard deviations are reported.

**Figure 3:**
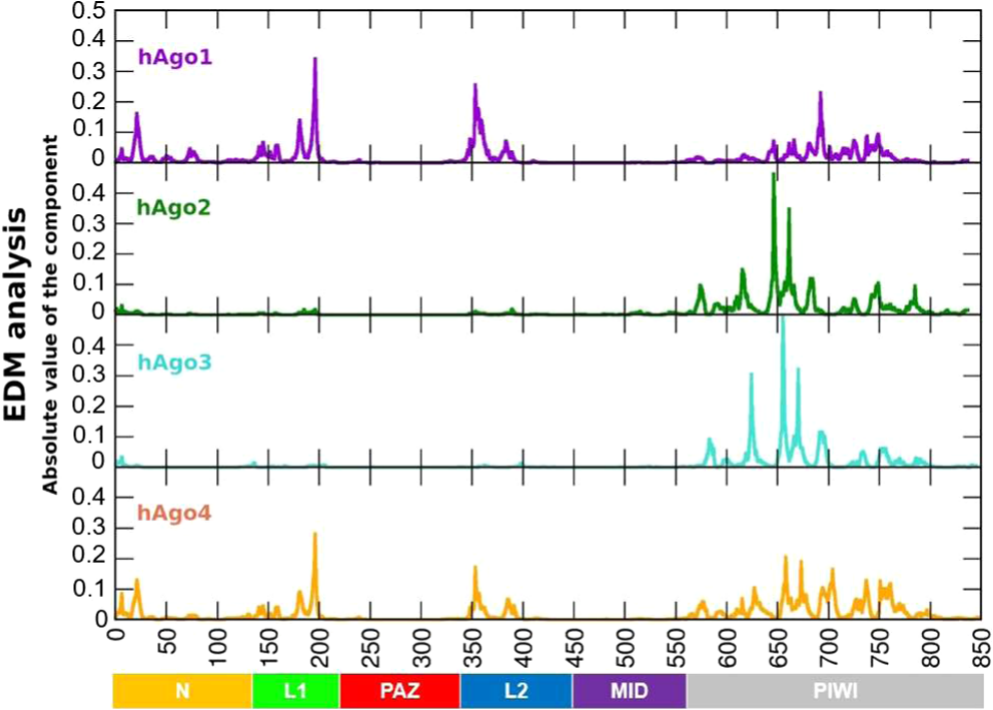
EDM analysis. Nonbonded interaction energy profiles of the most representative hAgos structure from MD simulations of the four hAgos in their apo states. The first centroid is taken from the cluster analysis performed on the full-length 1 μs trajectories (cluster #1 accounts for 37% (hAgo1), 32% (hAgo2), 32% (hAgo3), and 40% (hAgo4) of the total variance). The Beam domain residues are included in the N domain.

#### Interdomains Interaction Hotspots

The evaluation of the electrostatic bonds established among residues along the simulations provides insightful understanding of specific hotspots responsible for the differential modulation observed among the four hAgo paralogs. A detailed list of highly conserved interdomain hydrogen bonds and salt bridges along the 1 μs simulation time for the four proteins is reported in Tables 2a and 2b. Most of these stable interacting residues (present for at least 20% of the simulation time) bridge distal regions within the protein, thus accounting for their coordinated motions. Most of the interdomain interactions hotspots are common to all isoforms and help maintain the native protein fold. However, the number of bonds that stabilize hAgo4 isoform is larger than the others (see Tables 2a and 2b, Tables S4–S11), featuring a stronger interaction network involving both adjacent and distal domains.

**2A tbl2a:** Interdomain Hydrogen Bonds of hAgo apo conformations: **A**. Most meaningful hydrogen bonds found in MD Simulations of hAgos in the apo state (present in >20% trajectory frames in at least one isoform)[Table-fn t2afn1]

**RESIDUE A**		**RESIDUE B**	
**amino acid**	**hAgo domain**	**amino acid**	**hAgo domain**
**K266**	**PAZ**	**Q350**	**L2**
**T735**	**PIWI**	**R411**	**L2**
**H849**	**PIWI**	**D537**	**MID**
**Q350**	**L2**	**V347**	**PAZ**
**L419**	**L2**	**I577**	**MID**
**I346**	**PAZ**	**Q228**	**L1**
**L413**	**L2**	**G733**	**PIWI**
**L579**	**PIWI**	**S417**	**L2**
**R812**	**PIWI**	**D537**	**MID**
**A372**	**L2**	**S727**	**PIWI**
**R411**	**L2**	**Q780**	**PIWI**
**S796**	**PIWI**	**G433**	**L2**
**W435**	**L2**	**T794**	**PIWI**
**N575**	**MID**	**V791**	**PIWI**
**R714**	**PIWI**	**D218**	**L1**
**N576**	**MID**	**L419**	**L2**
**R411**	**L2**	**D737**	**PIWI**
K739^#^	PIWI	D407	L2

a Amino acid numbering corresponds to hAgo2 X-ray crystal structure (PDB ID: 4Z4C). **Bold**: hydrogen bonds conserved among the four hAgo isoforms. ^#^hydrogen bond exclusive of hAgo2.

**2B tbl2b:** Interdomain Hydrogen Bonds of hAgo apo conformations: **B.** Most meaningful salt bridges found in MD simulations of hAgos in the apo state (present in >20% trajectory frames in at least one isoform)[Table-fn t2bfn1]

**RESIDUE A**		**RESIDUE B**	
**amino acid**	**hAgo domain**	**amino acid**	**hAgo domain**
**D537**	**MID**	**H816**	**PIWI**
**D218**	**L1**	**R714**	**PIWI**
**E261**	**PAZ**	**R196**	**L1**
**D537**	**MID**	**R812**	**PIWI**
**D737**	**PIWI**	**R411**	**L2**
**D218**	**L1**	**R375**	**L2**
**E236**	**PAZ**	**K226**	**L1**
**D851**	**PIWI**	**K509**	**MID**
**D358**	**L2**	**K525**	**MID**
**E261**	**PAZ**	**K354**	**L2**
**D605**	**PIWI**	**K355**	**L2**
**E673**	**PIWI**	**R179**	**L1**
D499*	MID	K820	PIWI
E122*	NTER	K266	PAZ
E531*	MID	K820	PIWI
D407^#^	L2	K739	PIWI

a Amino acid numbering corresponds to hAgo2 X-ray crystal structure (PDB ID: 4Z4C). **Bold**: Salt bridge conserved among the four hAgo isoforms. *Salt bridge found in all isoforms but hAgo2. ^#^Salt bridge exclusive of hAgo2.

Our analysis globally reveals that (1) in hAgo2 and hAgo3, the PIWI domain interacts more strongly with the L2 domain than with MID, while the number of hydrogen bonds between L2-PIWI and MID-PIWI are comparable in hAgo1 and hAgo4; (2) L2-MID interplay is more pronounced in hAgo4 compared to the other paralogs; (3) a significant pattern of interaction between N and PAZ domains is observed in hAgo4 and, to a lesser extent, in hAgo1; (4) in hAgo4, the PIWI domain mediates a larger number of both inter- and intradomain bonds, reinforcing its role as stabilizing core.

Interestingly, in addition to conserved contacts mainly involved in stabilizing the tertiary fold, we identified several interactions within disordered regions bridging adjacent domains, that could inform on local and transient conformational rearrangements (Tables 2a, 2b, and S4–S11). Among the most significant findings, the N domain engages several interactions with PAZ in hAgo1 (E120-K274/R275/K276), hAgo3 (E114-K267/R281) and hAgo4 (E112-K256/K268/R270, D115-R267) (see Tables 2a and 2b). Notably, hAgo2 lacks these stabilizing contacts, which may contribute to its higher flexibility in this region. On the other hand, in hAgo2, the N domain establishes multiple contacts with L2 (E76-Y393, R90/K91-E396), while in hAgo1 (R88-E394) and hAgo3 (R83-E397) only single contacts are observed; no interactions are seen between these two domains in hAgo4.

In hAgo1 and hAgo4, PAZ flexibility is reduced due to the more persistent electrostatic interaction between PAZ and L2 domains. Specifically, the interaction between E259_hAgo1_ (E251_hAgo4_) and K352_hAgo1_ (K344_hAgo4_) occurs with persistence rates of 28% and 40%, respectively. Conversely, higher PAZ mobility is reflected by lower persistence rates in hAgo3 (E262-K355, 24%) and particularly in hAgo2 (E261-K354, 20%). It is worth noting that the interaction established by D407 in L2 and K739 in PIWI is solely reported for hAgo2 (Tables 2a and 2b), where it connects β strands at the bilobe junction, thus making these residues at the interface of different domains good candidates for mediating interlobe motions.

In hAgo1, we observe enhanced coordination between the PIWI and MID domains driven by interactions involving the flexible L5 loop (aa 822–832) of PIWI. Key interactions include D497-S826/K818, D535-R810. Similar interactions are partially observed in the other isoforms suggesting differential modulation of PIWI-MID coupling across the isoforms, specifically: D537-R812 (hAgo2), D538-H817 (hAgo3), D529-H818 (hAgo4) (Tables S4–S11).

A focus on L2 helix-7 (α7, aa 358–369 in hAgo2), a key regulator of protein activity, demonstrates a common interaction pattern across all the isoforms. One major salt-bridge between D358 and K525 (hAgo2) in the MID domain is consistently present. On the other side, helix-7 interacts with L1 (R194_hAgo1_). Additionally, two lysines (K352 and K353 in hAgo2) link the helix with PAZ and PIWI domains. It is important to note that α7-hAgo2 contains an arginine, R366, pointing into the RNA-cleft, while this position is occupied by a lysine in the other paralogs, leading to reduced steric hindrance. None of the amino acids in the catalytic tetrad interacts with neighboring protein residues. It is important to remind that the structural models of unbound human Argonautes simulated in the present study are derived from their RNA-bound crystallographic counterparts, therefore significant relaxation of the protein structures occurs during the initial phase of the MD simulation and this accommodation may depend on the nature/length of the bound RNAs. To gain insights into this aspect, we performed a comparative analysis with the RNA-bound states.

#### hAgo-miR-20a Complexes: RNA Modulation of Conformational Dynamics

The functional dynamics between the various domains, along with the breathing motion within the protein, are essential for defining the spatial configuration of the central channel that accommodates the RNA. This channel undergoes significant structural changes that facilitate key processes, including the initial binding of the miRNA duplex, the ejection of the passenger strand, the target recognition and pairing, and ultimately the degradation of mRNA. These sequential steps require multiple functional conformations of the protein.

While the apo-state dynamics of the isoforms alone cannot fully explain the structural changes required for these processes, investigating intrinsic differences in the RNA channel dynamics can provide insights into the distinct functional roles of these proteins. To explore this aspect, we calculated the RNA-binding pocket volume as the weighted average of the most representative conformations, identified through a cluster analysis of the conserved residues within the channel across the four isoforms (see Methods). Once again, despite the high degree of similarity (95.3%) between hAgo1 and hAgo2 in the RNA binding pocket structure, hAgo2 displays a distinct behavior. Consistent with its greater flexibility and plasticity, hAgo2 has a significantly larger RNA-binding channel compared to the other isoforms (17,287 Å^3^ in hAgo2 compared to 13,959 Å^3^ in hAgo1, 11,855 Å^3^ in hAgo3, and 13,686 Å^3^ in hAgo4). To assess the impact of RNA binding on the functional dynamics of the isoforms, we simulated the four proteins in the presence of a 21-*nt* miRNA with the nucleotide sequence of miR-20a. Overall, the presence of RNA (Table S2) has a negligible effect on hAgo2 and hAgo3, whereas its impact is more pronounced for isoforms hAgo1 and hAgo4, though with opposite outcomes. In hAgo1, RNA binding leads to increased conformational variability, whereas in hAgo4, it stabilizes the initial conformation. Domain-specific RMSD analysis reveals that the MID domain, where the 5′ end of the miRNA is anchored, undergoes a significant conformational response to RNA binding across all isoforms. Specifically, we observe a slight increase in RMSD for hAgo2 and hAgo3, while hAgo1 and hAgo4 show a reduction. We then use the distance fluctuation (DF) analysis to investigate how the nucleic acid modulates the protein’s internal dynamics and the coordination between its domains. DF is a technique that uses the evaluation of pairwise amino acids distances along the simulation time to disclose correlated motions between distal structural units. In the apo forms, DF matrices reveal overall similar profiles across isoforms, with PAZ showing higher flexibility compared to the rest of the protein, while the PIWI-MID block remains rigid. hAgo2 and hAgo3 display overlapping profiles, whereas hAgo1 shows enhanced coordination between the N and the MIDI-PIWI lobe. In contrast, hAgo4 exhibits the weakest coordination between PIWI and MID dynamics, in line with the interaction pattern described above (Figure 4). Upon RNA binding, more pronounced differences in internal dynamics emerge across the four isoforms. As expected, RNA increases overall protein rigidity, particularly reducing PAZ mobility as it becomes more anchored to the bilobed structure. Interestingly, hAgo2 is the least affected by RNA binding, maintaining relative flexibility between the N and PAZ domains, despite slight stiffening of a flexible loop-helix-loop region of PAZ (aa 220–250). Furthermore, RNA binding uniquely reduced the coordination between the PIWI catalytic subunit and MID in hAgo2. hAgo4, once again stands out as the most distinctive isoform: t is the only protein that shows a significant degree of decoupling between the dynamics of N and the MID-PIWI subunits. Interestingly, unlike hAgo2, RNA binding partially restores the dynamic coordination between PIWI and MID that was absent in the apo form.

**Figure 4:**
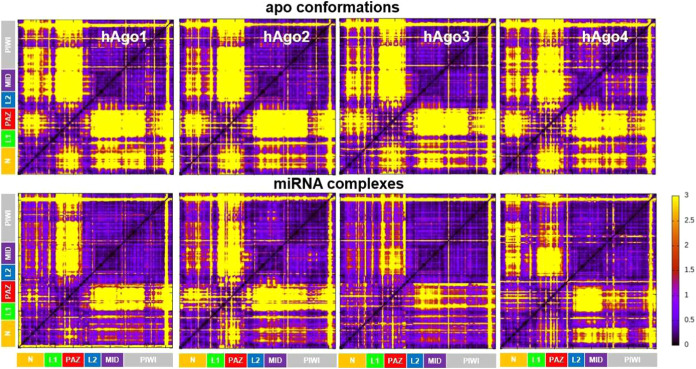
Distance Fluctuations. DF matrices of the four hAgo isoforms in their unbound state (top) and in complex with miRNA oligonucleotides (bottom). Yellow and purple areas in the matrices are associated with flexible and rigid regions, respectively. Protein domains are colored as in Figure 1. The Beam domain residues are included in the N domain. The color bar on the right reports the magnitude (Å^2^) of the fluctuations.

Upon RNA binding, a distinctive response of the local flexibility/adaptation of the proteins can be observed as well. Argonaute RNA channel itself shows a well-defined accommodation of the 21-*nt* miR-20a. Indeed, the comparison of the RMSFs of the RNA binding cleft displays a clear differential mobility of some regions along the protein for hAgo2. Higher fluctuation is observed at the PAZ and MID domains, with significant contributions from residues 220–222, 266–269, 277–280, and 308–316 of PAZ, and 521–533 and 545–566 of MID (Figure S5). RNA binding does not stiffen protein domains, rather its anchoring at the 5′ and 3′ confers enhanced flexibility to interacting fragments. This increased flexibility may promote transient states, which align with a more efficient RNA binding turnover mechanism.

The modulation of protein dynamics is reflected in the variation of nonbonded interaction energies between the protein and the miRNA. Overall, the nucleic acid exhibits weaker interactions with hAgo2 and hAgo3, with significantly lower interaction energies (416.0 and 403.5 kcal mol^–1^, respectively) compared to hAgo1 and hAgo4 (441.9 and 433.0 kcal mol^–1^, respectively). This trend is consistent with the reduced number of protein-RNA contacts in hAgo2 and hAgo3 compared to hAgo4 (Figure S6). hAgo1 presents a dual distribution, once again showing an intermediate behavior between the two catalytically active isoforms and hAgo4. This difference is primarily driven by the PIWI domain, which exhibits significantly stronger binding to the miRNA in hAgo4 (−147.5 kcal mol^–1^) compared to hAgo1 (−113.5 kcal mol^–1^), with hAgo2 showing intermediate binding (−131.0 kcal mol^–1^). Consistent with the dynamic data, the PAZ domain also displays a variable contribution to RNA binding, as in the case of hAgo4, where the significantly lower energy of interaction suggests a reduced efficiency in anchoring the nucleic acid compared to the other isoforms.

#### Effect of RNA Binding on Interaction Patterns

The variability in RNA binding energies observed across hAgo isoforms reflects differences in their ability to recognize and stabilize the guide-target RNA complex, this would be particularly important in the seed region where initial RNA pairing occurs. These interactions are further modulated by internal water networks, specifically in two internal pockets, termed LAKE1 and LAKE2.^26^ Upon guide RNA binding, these pockets form dynamic links between key regions stabilizing the L2, MID and PIWI domains in a conformation primed for target mRNA recognition. Survival probability analysis of water molecules in both LAKE pockets, comparing the apo- and miRNA-bound forms, confirms that nucleic acid binding stabilizes these pockets, leading to increased water residence times (Figure S7). Interestingly, while in the apo form, the water dynamics in both LAKE pockets are very similar for all four proteins, the presence of miRNA induces distinct isoform-specific differences, particularly in LAKE1. In hAgo2, the catalytically active isoform, RNA binding has minimal impact on water residence time in LAKE1. In contrast, hAgo4 exhibits a smaller pocket with reduced water mobility. hAgo1 and hAgo3 display intermediate behaviors, suggesting that maintaining a certain degree of flexibility in LAKE1 may facilitate conformational adaptation at the seed-binding site, enabling efficient guide-target pairing.

The distinct water dynamics in LAKE pockets upon RNA binding are mirrored in the differential interdomain stabilization observed across the four hAgo isoforms (Tables 2a, 2b, and S4–S11). In hAgo2, the guide RNA destabilizes only the structural clamp between MID and PIWI domains (namely, residues D537-R812/H816). Conversely, hAgo1 and hAgo4 miRNA binding induces more pronounced alterations in the interaction networks compared to their apo forms, suggesting a reduced conformational stability. hAgo3, like hAgo2, exhibits minimal changes upon miRNA binding, reflecting a shared tendency among catalytically active isoforms to retain flexibility and accessibility for nucleic acids recognition and binding.

#### Allosteric Propagation upon miRNA Binding

Since the four isoforms of the protein function redundantly, modulated by the presence of different miRNAs, a key question is whether allosteric regulation mechanisms can contribute to their functional differentiation. To identify allosteric hotspots and communication pathways activated upon miRNA binding, we combined the Gaussian Network Model (GNM) and the Markovian stochastic model^59,60^ with the analysis of efficient communication pathways.^61^ Our investigation focused on perturbation pathways linking key regulatory domains: the MID and PAZ domains, which bind the 5′ and 3′ ends of the miRNA respectively, and the N domain, which interfaces with critical regulatory proteins such as HSP90 and GW182.^10^ The analysis (see Methods and SI) was performed on representative structures obtained via cluster analysis, covering at least 80% of conformational variability. We aimed to identify the most recurrent allosteric hotspots involved in the most efficient communication pathways, assumed to correspond to key residues transmitting allosteric signals across the protein.

Our results show that allosteric communication between the MID and N domains differs across isoforms. In hAgo1 and hAgo4, a greater number of conserved hotspot residues are maintained between the apo and RNA-bound states, suggesting limited modulation of allosteric communication upon RNA binding (Table S12). In contrast, hAgo2 and hAgo3 exhibit enhanced MID–N domain communication efficiency in the bound state, as indicated by shorter commute times, whereas hAgo1 and hAgo4 show the opposite trend (Table S13). Across all isoforms, RNA binding promotes a redistribution of hotspots, increasing their occurrence in the MID domain at the expense of the PIWI domain (Table S12, Figure S8). Notably, residues Q545 and I567 in the MID domain of hAgo2 are highly conserved hotspots in the RNA-bound state across all isoforms. In hAgo2, Q545 (corresponding to Q543, Q546 and Q537 in hAgo1, hAgo3 and hAgo4, respectively) forms hydrogen bonds with the 5′-terminal phosphate of the miRNA (Figure S9), a critical interaction observed in the X-ray structure of hAgo2 (PDB ID4F3T) and essential for guide RNA positioning and target RNA cleavage.^23,62^ Furthermore, the hotspots N43 and N216 in the N domain (hAgo2 numbering) emerge as key hotspot residues that are conserved in both apo and bound states across isoforms. Experimental mutations of these residues to alanine have been shown to impair miRNA unwinding and reduce passenger RNA cleavage.^2^

The analysis of the allosteric hotspots distribution between the PAZ and N domains further distinguishes catalytically active (i.e., hAgo2 and hAgo3) from inactive (i.e., hAgo1 and hAgo4) isoforms. Specifically, hAgo1 and hAgo4 undergo an inward shift of key residues toward the RNA binding channel in the presence of miRNA, in contrast to their apo states (Figure S10). Conversely, hAgo2 and hAgo3 display a partial outward shift. These macroscopic shifts result from an increase in key residues within the N domain at the expense of L1 in hAgo2 and hAgo3 between the apo and bound forms, while the opposite trend is observed in hAgo1 and hAgo4 (Table S14). Consistent with the MID-N allosteric patterns, the shortest communication pathways between the PAZ and N domains indicate enhanced communication efficiency in hAgo2 and hAgo3 upon miRNA binding. Conversely, communication is attenuated in hAgo1 and hAgo4 under the same conditions (Table S15 and Figure S9).

## Discussion

MD simulations have unveiled subtle, yet significant dissimilarities among closely related human Argonautes paralogs, shedding light on their functional divergence in RNA silencing. While these proteins share significant structural similarities, minor differences in their conformational dynamics seem to fine-tune the sequence-structure–function relationships typical of protein paralogs. Microsecond-scale MD simulations of the four isoforms in their apo states revealed that, besides shared interdomain assembly dynamics, the enhanced structural flexibility of hAgo2, and, to a lesser extent, hAgo3, results in significant interlobe ‘breathing’ motions, which are essential for slicing activity as previously reported. This dynamic behavior is linked to the increased flexibility of the PAZ domain in hAgo2, which is crucial for recognizing miRNAs. The ability of hAgo2 to accommodate diverse RNA substrates through conformational selection may explain its unique role in RNA silencing, distinguishing it from other isoforms. This increased flexibility likely enables hAgo2 to handle a broader range of RNA targets, contributing to its dominant function in the process.

An alternative use of PCA, focusing on secondary components, unveiled hidden variations in local conformational mobility particularly within the L2 and PIWI subunits. These rearrangements, coupled to the PAZ–PIWI relative motion, capture dynamics features that are intrinsic to hAgo2 and hAgo3. Although few interactions differentiate the isoforms, they are localized in mobile interdomain connecting protein regions, with the most significant impact observed at the nucleic acid binding cleft, where they influence both accessibility and structural adaptability of the RNA channel. This dynamic picture is also mirrored in a well-defined energy signature of the protein paralogs. Our results show that all four human isoforms rely on a small set of highly conserved stabilization determinants, primarily located within the PIWI domain, where catalytic activity is exerted, ensuring structural stability essential for function. However, in the less catalytically active isoforms, hAgo1 and hAgo4, additional accessory regions contribute to a distinctive energy fingerprint. We speculate that these accessory regions may be linked to a more regulatory and modular nature of these isoforms, offering a potential regulatory role beyond the catalytic function of hAgo2 and hAgo3.

These functional distinctions are underpinned by a set of isoform-specific residue–domain interactions that fine-tune conformational behavior and RNA processing. For instance, the coordination between N and L2 domains (namely, via interactions between residues E76-Y393 and R90/K91-E396 of hAgo2) likely modulates N-domain displacement, directly impacting RNA processing. Similarly, enhanced mobility at the PAZ-L2 interface (namely, mediated by a hydrogen bond between E261 and K354 in hAgo2) reflects coordinated motion of structural elements such as the α7 helix, which is important for RNA pairing and stabilization.

The binding of a 21*-nt* miRNA induces isoform-specific conformational adjustments, with hAgo2 exhibiting enhanced pocket accessibility and increased flexibility in RNA-interacting regions. Reduced mechanical coordination between PIWI and MID domains, coupled with greater flexibility in the N and PAZ domains and lower nonbonded interaction energies for hAgo2 and hAgo3, supports transient and adaptable RNA interactions critical for slicing activity. In hAgo2, this transient RNA binding allows the guide RNA to remain loosely held, facilitating the conformational changes required for catalysis. RNA binding does not rigidify protein domains, rather, it enhances the flexibility of interacting regions, promoting transient states aligned with efficient RNA turnover. Conversely, hAgo4’s tighter structural coordination, compact morphology, and rigid conformation likely constrain the conformational freedom needed for effective slicing, while hAgo1 and hAgo3 display intermediate behavior.

Further supporting these differences, distinct water dynamics were observed in the LAKE pockets, key internal hydration cavities known to stabilize RNA-protein complexes. In hAgo2, shorter water residence times in LAKE1 suggest that maintaining flexibility in this pocket supports conformational adaptation at the seed-binding site, thereby facilitating RNA-target pairing. In catalytically active isoforms, such protein flexibility also contributes to efficient allosteric signal propagation. Analyses of allosteric communication pathways between either the 5′ or 3′ ends of the miRNA and key residues in the N domain, identified as a critical regulatory hub for RNA binding due to its interactions with a regulatory interactome, reveal clear isoform-dependent. In hAgo2 and hAgo3, RNA binding enhances communication efficiency along both the N-MID and N-PAZ pathways, whereas hAgo1 and hAgo4 exhibit limited changes compared to their unbound forms. These findings suggest a finely tuned trade-off between the conservation of apo-to-bound allosteric hotspots and the structural transitions necessary for functional adaptability.

## Conclusion

In conclusion, our results frame up a highly dynamic system in which the balance between structural rigidity and flexibility is crucial for the functional specialization of human Argonaute isoforms. hAgo2 emerges as uniquely adapted, with optimized flexibility and structural plasticity that allow dynamic transitions necessary for efficient RNA binding and slicing. In contrast, hAgo4 adopts a more rigid conformation, consistent with its reduced catalytic activity, while hAgo1 and hAgo3 exhibit an intermediate behavior, highlighting the need of further studies to refine our understanding of their structural dynamics.

These findings underscore the significance of conformational dynamics in modulating the RNA silencing efficiency of human Argonautes, demonstrating that even subtle structural differences can trigger distinct dynamic responses with crucial functional outcomes. Our study provides a framework in which the functional divergence of Argonautes is closely linked to their capacity for flexible and dynamic interactions with nucleic acids, enabling more effective RNA silencing mechanisms. This work contributes to advancing our understanding of the molecular regulation of RNA-mediated gene silencing, offering insights into the broader implications for gene regulation and new perspectives for therapeutic strategies targeting RNA interference pathways.

## Supplementary Material



## Data Availability

Atomic coordinates of Argonaute proteins in their unbound (apo-hAgos) and RNA-bound states (hAgos-miR-20a), along with scripts and related data, are accessible through a Zenodo repository (http://doi.org/10.5281/zenodo.14844878).

## Notes

hAgo1–4 sequence similarity (Table S1); average RMSD and Gyration Radius *per* isoform (Table S2); inter- and intramolecular interactions (Tables S3–S11); list of residues in MID-N and PAZ-N communication pathways (Tables S12–S15); hAgo1–4 sequence alignment (Figure S1); RMSD and RMSF evolution upon RNA binding (Figures S2, S5); Principal Component Analysis and Hydrogen bonds *per* domain of hAgo isoforms in the apo state (Figures S3–S4); RNA-hAgos contacts distribution (Figure S6); water survival probability changes upon RNA-binding (Figure S7); structural mapping of MID-N and PAZ-N communication pathways (Figures S8–S10) (PDF)
