# Supplementary material for: Conformational Dynamics of hAgo2 Silencing: Decoding Functional Divergence across Human Argonaute Paralogs
Source: J Chem Inf Model. 2025 Jun 5;65(18):9528–40. doi: 10.1021/acs.jcim.5c00194 (PMC12458702; doi:10.1021/acs.jcim.5c00194)
Supplement: Supplementary file 1 [file ci5c00194_si_001.pdf]

Supporting Information

Conformational Dynamics of hAgo2 Silencing: Decoding Functional Divergence Across Human Argonaute Paralogs

Antonella Paladino [a,#,*] Andrea Catte [b,#], Jorge Franco [a], Elisabetta Moroni [c], Silvia Rinaldi [b,*]

[a] Institute of Biostructures and Bioimaging, IBB-CNR, Via Pietro Castellino 111, 80131 Napoli, Italy

[b] Institute of Chemistry of OrganoMetallic Compounds, ICCOM-CNR, Via Madonna del Piano 10, 50019 Sesto Fiorentino, Firenze, Italy

[c] Institute of Chemical Sciences and Technologies, SCITEC-CNR, via Mario Bianco 9, 20131 Milano, Italy.

Multiple sequence alignments of human AGO isoforms

Multiple sequence alignments (MSAs) of the four human AGO (hAGO1-4) isoforms were performed by ClustalW [1,2] using the MultiSeq plugin of VMD (http://www.ks.uiuc.edu/Research/vmd/plugins/multiseq/) [3], verifying the absence of gaps in important secondary structure regions. The MSA of the full length hAGOs was in good agreement/coincided with the one reported by Park et al. in Figure S3 of the Supporting Information of their 2019 article [4] (**Figure S1**). The sequence identities and similarities among different protein domains, defined as in Nakanishi 2022 [5] review (**Figure S1**), were calculated from MSAs with EMBOSS Stretcher [6] and reported in **Table S1**.

Table S1. Sequence identities (similarities) of different hAgo isoforms are reported as percentages in the table.

Domain	hAgo	hAgo1	hAgo3	hAgo4
All	hAgo1	-	84.1 (91.0)	82.6 (90.1)
	hAgo2	82.6 (91.4)	79.5 (88.6)	77.6 (87.6)
	hAgo3	84.1 (91.0)	-	80.8 (88.6)
Beam	hAgo1	-	60.8 (70.6)	60.8 (70.6)
	hAgo2	52.8 (64.2)	46.3 (55.6)	45.3 (56.6)
	hAgo3	60.8 (70.6)	-	62.2 (71.1)
N	hAgo1	-	70.1 (82.8)	70.9 (88.4)
	hAgo2	75.6 (91.9)	70.1 (86.2)	72.1 (91.9)
	hAgo3	70.1 (82.8)	-	69.0 (87.4)
	hAgo1	-	73.5 (78.6)	81.1 (85.6)
L1	hAgo2	76.9 (87.9)	67.3 (77.6)	74.4 (86.7)
	hAgo3	73.5 (78.6)	-	69.4 (74.5)
PAZ	hAgo1	-	89.8 (95.8)	91.5 (98.3)
	hAgo2	87.3 (94.9)	81.4 (92.4)	83.9 (93.2)
	hAgo3	89.8 (95.8)	-	86.4 (94.9)
L2	hAgo1	-	78.6 (90.8)	80.0 (90.0)
	hAgo2	77.6 (89.8)	74.5 (85.7)	71.0 (84.0)
	hAgo3	78.6 (90.8)	-	76.0 (86.0)
MID	hAgo1	-	94.7 (98.5)	92.4 (97.7)
	hAgo2	87.9 (97.0)	86.4 (95.5)	85.6 (94.7)
	hAgo3	94.7 (98.5)	-	88.6 (96.2)
PIWI	hAgo1	-	90.8 (96.1)	83.2 (88.7)
	hAgo2	89.4 (94.0)	90.8 (95.7)	82.2 (88.0)
	hAgo3	90.8 (96.1)	-	87.0 (91.4)
RNA binding pocket	hAgo1	-	97.3 (97.3)	96.6 (97.3)
	hAgo2	90.6 (95.3)	90.6 (95.3)	90.6 (95.3)
	hAgo3	97.3 (97.3)	-	95.3 (96.0)

Table S2. A. Root Mean Square Deviation *per* domain. RMSD (Å) is averaged along the 1 μs MD trajectory. The errors are standard deviations.

APO	N-ter	L1	PAZ	L2	MID	PIWI
**hAgo1**	2.9 ± 0.8	2.2 ± 0.4	5.0 ± 1.7	2.3 ± 0.5	2.7 ± 0.8	3.6 ± 0.4
**hAgo2**	3.5 ± 0.7	2.2 ± 0.3	6.7 ± 1.6	2.9 ± 0.5	3.5 ± 0.9	3.7 ± 0.5
**hAgo3**	3.8 ± 1.0	2.5 ± 0.3	5.5 ± 1.7	2.5 ± 0.5	2.5 ± 0.6	3.6 ± 0.4
**hAgo4**	3.7 ± 0.9	2.0 ± 0.3	5.6 ± 1.3	2.8 ± 0.4	3.5 ± 0.8	3.9 ± 0.4

Bound	N-ter	L1	PAZ	L2	MID	PIWI
**hAgo1**	6.1 ± 0.7	3.9 ± 0.6	4.0 ± 1.0	3.5 ± 0.4	2.3 ± 0.5	4.5 ± 0.5
**hAgo2**	3.2 ± 0.5	2.8 ± 0.5	5.2 ± 1.0	2.8 ± 0.5	3.2 ± 0.7	3.2 ± 0.6
**hAgo3**	2.6 ± 0.4	2.2 ± 0.2	3.1 ± 0.6	2.0 ± 0.3	1.9 ± 0.4	2.5 ± 0.3
**hAgo4**	3.1 ± 0.6	1.8 ± 0.2	3.0 ± 0.6	2.2 ± 0.3	2.3 ± 0.5	3.2 ± 0.4

B. Radius of gyration (Rg) is averaged along the 1 μs MD trajectory. The errors are standard deviations (SDs).

hAgo	Rg (Å)	SD
**hAgo1**	31.56	0.48
**hAgo2**	31.44	0.48
**hAgo3**	31.05	0.40
**hAgo4**	30.95	0.33

Table S3. Non-bonded interactions (electrostatic and van der Waals) between protein domains of hAgo isoforms in the presence and in the absence of 21-nt miR-20a.

APO							
Protein domains	Isoform	Coulombic		Lennard-Jones		Total	
		Average	Error	Average	Error	Average	Error
		kcal mol^{-1}					
PAZ-L1	hAgo1	-14.8	2.6	-39.9	4.1	-54.7	4.9
	hAgo2	-15.2	3.2	-39.9	4.6	-55.1	5.6
	hAgo3	-12.3	2.4	-32.8	4.3	-45.1	4.9
	hAgo4	-11.1	2.7	-32.2	4.0	-43.3	4.8
PAZ-L2	hAgo1	-7.0	1.6	-11.4	2.5	-18.4	3.0
	hAgo2	-6.8	1.6	-11.8	2.5	-18.6	3.0
	hAgo3	-6.9	1.5	-12	2.3	-18.9	2.7
	hAgo4	-7.2	1.7	-11.2	2.7	-18.4	3.2
L2-MID	hAgo1	-9.4	1.7	-64.4	5.0	-73.8	5.3
	hAgo2	-10.9	1.9	-63.0	6.9	-73.9	7.2
	hAgo3	-12.7	2.4	-36.4	4.2	-49.1	4.8
	hAgo4	-12.9	2.1	-139.3	6.3	-152.2	6.6
N-PIWI	hAgo1	-14.3	1.6	-99.4	6.4	-113.7	6.6
	hAgo2	-19.3	2.4	-94.7	6.5	-114	6.9
	hAgo3	-14.5	1.8	-106.1	5.7	-120.6	6.0
	hAgo4	-16.2	2.3	-101.3	6.8	-117.5	7.2
L2-PIWI	hAgo1	-10.0	1.8	-134.6	6.2	-144.6	6.5
	hAgo2	-11.3	1.8	-126.7	7.0	-138	7.2
	hAgo3	-12.3	3.9	-129.1	7.7	-141.4	8.6
	hAgo4	-12.6	2.9	-131.8	7.8	-144.4	8.3
MID-PIWI	hAgo1	-15.7	5.5	-107.1	13.0	-122.8	14.1
	hAgo2	-11.8	1.7	-88.0	6.4	-99.8	6.6
	hAgo3	-17.6	3.4	-104.6	10.0	-122.2	10.6
	hAgo4	-13.5	4.8	-91.6	16.3	-105.1	17.0
N-PAZ	hAgo1	-2.9	2.3	-8.8	6.2	-11.7	6.6
	hAgo2	-0.7	1.5	-0.7	1.9	-1.4	2.4
	hAgo3	-2.4	2.4	-10.4	6.6	-12.8	7.0
	hAgo4	-6.9	3.3	-16.2	7.8	-23.1	8.5

RNA-BOUND							
Protein domains	Isoform	Coulombic		Lennard-Jones		Total	
		Average	Error	Average	Error	Average	Error
		kcal mol^{-1}					
PAZ-L1	hAgo1	-11.4	2.9	-36.9	3.5	-48.3	4,5
	hAgo2	-13.9	3.5	-39.1	4.4	-53	5,6
	hAgo3	-13	2.6	-31.2	3.7	-44.2	4,5
	hAgo4	-10.1	1.5	-30.5	3.9	-40.6	4,2
PAZ-L2	hAgo1	-7.1	1.5	-12.1	2.8	-19.2	3,2
	hAgo2	-6.4	1.8	-13.3	2.7	-19.7	3,2
	hAgo3	-6.6	1.5	-13.3	3	-19.9	3,34
	hAgo4	-6.8	1.4	-12.1	2.5	-18.9	2,9
L2-MID	hAgo1	-11.2	1.8	-63.1	4.9	-74.3	5,2
	hAgo2	-10.5	1.8	-58.5	4.3	-69	4,7
	hAgo3	-13.2	2.6	-37.1	4.7	-50.3	5,4
	hAgo4	-14.4	2.1	-141.7	6.4	-156.1	6,7
N-PIWI	hAgo1	-12.5	2.1	-67.4	13.5	-79.9	13,7
	hAgo2	-22.7	3.5	-121.2	17	-143.9	17,4
	hAgo3	-16.6	2.3	-117	10.3	-133.6	10,6
	hAgo4	-15.3	2.8	-104.4	31.5	-119.7	31,6
L2-PIWI	hAgo1	-10.3	2.3	-143.4	8.7	-153.7	9,0
	hAgo2	-12.1	2.1	-136.8	7	-148.9	7,3
	hAgo3	-9.7	2.4	-131.9	6.5	-141.6	6,9
	hAgo4	-9.5	1.8	-145.2	6.5	-154.7	6,7
MID-PIWI	hAgo1	-14.1	2.4	-93.6	9	-107.7	9,3
	hAgo2	-8.1	3.5	-81.8	6.3	-89.9	7,2
	hAgo3	-15	2.5	-99.3	8.1	-114.3	8,5
	hAgo4	-15.7	3	-100.3	8.4	-116	8,9
N-PAZ	hAgo1	-1	1.7	-8.1	6.1	-9.1	6,3
	hAgo2	-0.8	1.9	-3.8	3.9	-4.6	4,3
	hAgo3	-1.8	1.5	-14.1	3.7	-15.9	4,0
	hAgo4	-0.1	1.6	-3.9	2.6	-4	3,1

Table S4. Strongest hydrogen bonds (occurrence in trajectory > 20%) computed in molecular dynamics simulations of **hAgo1** in the apo state.

**RESIDUE A**		**RESIDUE B**		**Strength (%)**
**Amino acid**	**hAgo1 domain**	**Amino acid**	**hAgo1 domain**	
LYS264-Main-N	PAZ	GLN348-Side-OE1	L2	62.26
THR733-Main-N	PIWI	ARG409-Main-O	L2	58.21
SER794-Main-N	PIWI	GLY431-Main-O	L2	53.71
GLN348-Side-NE2	L2	VAL345-Main-O	PAZ	53.09
ILE344-Main-N	PAZ	GLN226-Main-O	L1	51.03
HIE847-Main-N	PIWI	ASP535-Main-O	MID	50.57
LEU411-Main-N	L2	GLY731-Main-O	PIWI	46.60
GLN417-Main-N	L2	ILE575-Main-O	MID	42.63
ASN442-Main-N	L2	ILE572-Main-O	MID	42.02
ARG409-Side-NH2	L2	GLN778-Side-OE1	PIWI	40.26
PHE440-Main-N	L2	ALA479-Main-O	MID	40.18
TYR441-Main-N	L2	ASN573-Main-O	MID	36.06
VAL577-Main-N	PIWI	ILE415-Main-O	L2	33.38
TRP433-Main-N	L2	THR792-Main-O	PIWI	31.09
ASN566-Side-ND2	MID	ASN442-Main-O	L2	29.26
ALA730-Main-N	PIWI	GLN430-Side-OE1	L2	27.81
ARG712-Side-NE	PIWI	ASP216-Side-OD2	L1	25.06
PHE231-Main-N	PAZ	PRO227-Main-O	L1	24.83
ASN573-Side-ND2	MID	VAL789-Main-O	PIWI	23.76
GLN439-Side-NE2	L2	ASP478-Main-O	MID	22.46
ALA370-Main-N	L2	SER725-Main-O	PIWI	21.93
ILE415-Main-N	L2	GLN580-Side-OE1	PIWI	20.70

Table S5. Strongest hydrogen bonds (occurrence in trajectory > 20%) computed in molecular dynamics simulations of **hAgo2** in the apo state.

**RESIDUE A**		**RESIDUE B**		**Strength (%)**
**Amino acid**	**hAgo2 domain**	**Amino acid**	**hAgo2 domain**	
LYS266-Main-N	PAZ	GLN350-Side-OE1	L2	61,78
THR735-Main-N	PIWI	ARG411-Main-O	L2	59,65
HIE849-Main-N	PIWI	ASP537-Main-O	MID	56,9
GLN350-Side-NE2	L2	VAL347-Main-O	PAZ	52,17
PHE442-Main-N	L2	ALA481-Main-O	MID	46,38
LEU419-Main-N	L2	ILE577-Main-O	MID	46,07
ILE346-Main-N	PAZ	GLN228-Main-O	L1	44,62
LEU413-Main-N	L2	GLY733-Main-O	PIWI	42,41
THR444-Main-N	L2	VAL574-Main-O	MID	41,5
LEU579-Main-N	PIWI	SER417-Main-O	L2	40,2
CYS352-Main-N	L2	ALA223-Main-O	L1	38,29
TYR225-Main-N	L1	GLN350-Main-O	L2	37,3
ARG812-Side-NH1	PIWI	ASP537-Side-OD2	MID	36,69
ALA372-Main-N	L2	SER727-Main-O	PIWI	36
ARG411-Side-NH2	L2	GLN780-Side-OE1	PIWI	35,55
HIE443-Main-N	L2	ASN575-Main-O	MID	35,24
SER796-Main-N	PIWI	GLY433-Main-O	L2	29,82
TRP435-Main-N	L2	THR794-Main-O	PIWI	27,69
LYS739-Side-NZ	PIWI	ASP407-Main-O	L2	26,7
ASN568-Side-ND2	MID	THR444-Main-O	L2	22,65
GLY755-Main-N	PIWI	GLN432-Main-O	L2	22,43

Table S6. Strongest hydrogen bonds (occurrence in trajectory > 20%) computed in molecular dynamics simulations of human **hAgo3** in the apo state.

RESIDUE A		RESIDUE B		Strength (%)
**Amino acid**	**hAgo3 domain**	**Amino acid**	**hAgo3 domain**	
LYS267-Main-N	PAZ	GLN351-Side-OE1	L2	60,08
THR736-Main-N	PIWI	ARG412-Main-O	L2	58,39
GLN351-Side-NE2	L2	VAL348-Main-O	PAZ	53,17
HIE850-Main-N	PIWI	ASP538-Main-O	MID	52,82
ARG412-Side-NH2	L2	GLN781-Side-OE1	PIWI	51,41
GLN420-Main-N	L2	ILE578-Main-O	MID	47,6
ARG813-Side-NH1	PIWI	ASP538-Side-OD2	MID	47,04
ILE347-Main-N	PAZ	GLN229-Main-O	L1	45,91
LEU414-Main-N	L2	GLY734-Main-O	PIWI	42,81
ASN576-Side-ND2	MID	VAL792-Main-O	PIWI	40,55
CYS353-Main-N	L2	ALA224-Main-O	L1	37,87
SER797-Main-N	PIWI	GLY434-Main-O	L2	35,61
TRP436-Main-N	L2	THR795-Main-O	PIWI	35,4
TYR226-Main-N	L1	GLN351-Main-O	L2	34,56
GLY422-Main-N	L2	GLN442-Main-O	MID	33,15
MET418-Main-N	L2	GLN583-Side-OE1	PIWI	31,88
ASN577-Side-ND2	MID	GLN420-Main-O	L2	28,56
ALA373-Main-N	L2	SER728-Main-O	PIWI	25,74
VAL580-Main-N	PIWI	MET418-Main-O	L2	23,27
ARG412-Side-NH2	L2	ASP738-Side-OD2	PIWI	21,23
ARG208-Side-NH1	L1	ILE741-Main-O	PIWI	20,52

Table S7. Strongest hydrogen bonds (occurrence in trajectory > 20%) computed in molecular dynamics simulations of human **hAgo4** in the apo state.

RESIDUE A		RESIDUE B		Strength (%)
**Amino acid**	**hAgo4 domain**	**Amino acid**	**hAgo4 domain**	
THR737-Main-N	PIWI	ARG403-Main-O	L2	59.13
LYS256-Main-N	PAZ	GLN340-Side-OE1	L2	55.95
GLN340-Side-NE2	L2	VAL337-Main-O	PAZ	53.68
GLN411-Main-N	L2	VAL569-Main-O	MID	50.14
LEU405-Main-N	L2	GLY735-Main-O	PIWI	49.14
HIE851-Main-N	PIWI	ASP529-Main-O	MID	47.68
VAL511-Main-N	MID	ALA444-Main-O	L2	46.14
SER798-Main-N	PIWI	GLY425-Main-O	L2	39.42
PHE434-Main-N	L2	ALA473-Main-O	MID	39.33
GLN478-Main-N	MID	ILE440-Main-O	L2	39.24
MET409-Main-N	L2	GLN574-Side-OE1	PIWI	39.06
ASN567-Side-ND2	MID	VAL793-Main-O	PIWI	37.15
ILE336-Main-N	PAZ	GLN218-Main-O	L1	35.79
TYR215-Main-N	L1	GLN340-Main-O	L2	35.69
ARG814-Side-NH1	PIWI	ASP529-Side-OD2	MID	35.51
ILE440-Main-N	L2	PRO476-Main-O	MID	34.97
TRP427-Main-N	L2	THR796-Main-O	PIWI	34.15
TRP443-Side-NE1	L2	GLY479-Main-O	MID	34.06
CYS342-Main-N	L2	ALA213-Main-O	L1	34.06
ALA436-Main-N	L2	ILE566-Main-O	MID	33.70
VAL445-Main-N	L2	PHE483-Main-O	MID	29.70
ASN568-Side-ND2	MID	GLN411-Main-O	L2	29.25
TYR435-Main-N	L2	ASN567-Main-O	MID	28.70
ALA362-Main-N	L2	SER729-Main-O	PIWI	25.70
ALA734-Main-N	PIWI	GLN424-Side-OE1	L2	25.07
ARG403-Side-NH2	L2	GLN782-Side-OE1	PIWI	23.25
ASN560-Side-ND2	MID	ALA436-Main-O	L2	22.71
ARG403-Side-NH2	L2	ASP739-Side-OD2	PIWI	22.16
TYR385-Side-OH	L2	PRO82-Main-O	NTER	21.53
GLN433-Side-NE2	L2	ASP472-Main-O	MID	21.44
ARG163-Side-NH1	L1	ASP383-Side-OD2	L2	21.07

Table S8. Strongest salt bridges (occurrence in trajectory > 20%) computed in molecular dynamics simulations of human **hAgo1** in the apo state.

RESIDUE A		RESIDUE B		Strength (%)
**Amino acid**	**hAgo1 domain**	**Amino acid**	**hAgo1 domain**	
ASP216	L1	ARG373	L2	99,77
ASP535	MID	HIE814	PIWI	80,14
ASP735	PIWI	ARG409	L2	78,46
GLU259	PAZ	ARG194	L1	75,10
ASP216	L1	ARG712	PIWI	74,03
GLU234	PAZ	LYS224	L1	67,38
GLU394	L2	ARG88	NTER	58,29
ASP389	L2	ARG171	L1	45,53
GLU120	NTER	ARG275	PAZ	28,27
GLU259	PAZ	LYS352	L2	28,04
ASP535	MID	ARG810	PIWI	23,30
ASP497	MID	LYS818	PIWI	20,63

Table S9. Strongest salt bridges (occurrence in trajectory > 20%) computed in molecular dynamics simulations of human **hAgo2** in the apo state.

RESIDUE A		RESIDUE B		Strength (%)
**Amino acid**	**hAgo2 domain**	**Amino acid**	**hAgo2 domain**	
ASP537	MID	HIE816	PIWI	98,09
ASP218	L1	ARG714	PIWI	92,98
GLU261	PAZ	ARG196	L1	78,57
ASP537	MID	ARG812	PIWI	77,88
ASP737	PIWI	ARG411	L2	66,44
ASP218	L1	ARG375	L2	62,40
GLU396	L2	ARG90	NTER	58,89
GLU236	PAZ	LYS226	L1	54,92
ASP851	PIWI	LYS509	MID	36,69
ASP358	L2	LYS525	MID	26,16
GLU673	PIWI	ARG207	L1	23,49
ASP407	L2	LYS739	PIWI	20,44

Table S10. Strongest salt bridges (occurrence in trajectory > 20%) computed in molecular dynamics simulations of human **hAgo3** in the apo state.

RESIDUE A		RESIDUE B		Strength (%)
**Amino acid**	**hAgo3 domain**	**Amino acid**	**hAgo3 domain**	
ASP538	MID	ARG813	PIWI	95,98
ASP538	MID	HIE817	PIWI	89,28
ASP738	PIWI	ARG412	L2	84,20
ASP219	L1	ARG715	PIWI	79,97
GLU262	PAZ	ARG197	L1	73,48
ASP219	L1	ARG376	L2	49,93
GLU237	PAZ	LYS227	L1	41,26
ASP852	PIWI	LYS510	MID	39,21
GLU674	PIWI	ARG180	L1	31,95
ASP219	L1	ARG762	PIWI	30,39
ASP359	L2	LYS526	MID	26,02
GLU262	PAZ	LYS355	L2	24,19
GLU397	L2	ARG83	NTER	22,64

Table S11. Strongest salt bridges (occurrence in trajectory > 20%) computed in molecular dynamics simulations of human **hAgo4** in the apo state.

RESIDUE A		RESIDUE B		Strength (%)
**Amino acid**	**hAgo4 domain**	**Amino acid**	**hAgo4 domain**	
ASP739	PIWI	ARG403	L2	83,11
ASP208	L1	ARG716	PIWI	79,56
ASP208	L1	ARG763	PIWI	67,30
ASP529	MID	ARG814	PIWI	61,22
ASP529	MID	HIE818	PIWI	59,58
ASP383	L2	ARG163	L1	59,22
GLU251	PAZ	ARG186	L1	52,41
ASP208	L1	ARG365	L2	45,59
GLU143	L1	LYS679	PIWI	41,60
GLU251	PAZ	LYS344	L2	40,24
GLU112	NTER	LYS256	PAZ	28,97
ASP853	PIWI	LYS501	MID	28,43
ASP348	L2	LYS517	MID	25,52
GLU226	PAZ	ARG216	L1	23,34
GLU523	MID	LYS822	PIWI	23,16
ASP597	PIWI	LYS345	L2	20,07

Table S12. List of top 15 residues of MID-N shortest communication pathways weighted over the most representative structures of the top 3 clusters of hAgo apo conformations and 21nt miR-20a complexes. The top 15 residues conserved between apo structures and 21nt miR-20a complexes of each isoform are in bold characters. Table cells of different protein domains are colored using the same color code of Figure 1. The Beam domain residues are included in the N domain. Residues of the PIWI domain are highlighted with a gray shade.

hAgo domain	hAgo1		hAgo2		hAgo3		hAgo4	
	apo	21nt	apo	21nt	apo	21nt	apo	21nt
N	**N41**	**N41**	**N43**	**N43**	**V160**	N35	N33	**D153**
	D161	**V204**	**V206**	L162	D164	Y93	**D153**	V154
L1	**V204**	**N214**	**N216**	**V206**	V207	**V160**	**V196**	**V196**
	**N214**	Q543	Y529	**N216**	**R208**	A162	N206	P519
MID	K531	L563	K533	Q545	**A210**	**R208**	A535	Q537
	**R712**	I565	A596	L565	**Q546**	**A210**	R716	L557
	**S750**	**R712**	R714	I567	L566	A531	R763	I559
	**Y788**	C749	**P762**	L715	**I568**	**Q546**	S765	A588
	**R790**	**S750**	S763	L750	D598	**I568**	I801	G671
PIWI	**S794**	R759	I799	S752	D670	R715	A805	Y792
	I797	**Y788**	A803	**P762**	V707	R762	**A808**	**A808**
	A804	**R790**	A806	Y790	A755	S764	**A812**	**A812**
	V807	**S794**	A810	R792	C794	Y791	**A815**	**A815**
	**A811**	**A811**	**A813**	S796	A811	R793	**H818**	**H818**
	**H814**	**H814**	H816	**A813**	A814	I800	Y859	V820

Table S13 MID-N shortest communication pathways and commute times of the most representative structure of cluster 1of hAgo apo conformations and 21-nt miR-20a complexes. The top 15 conserved residues are highlighted in red.

hAgo	sys	MID-N shortest communication pathway	Ct[a]
hAgo1	apo	Y527 -> K531 -> H814 -> A811 -> V807 -> A804 -> A801 -> I797 -> S761 -> R759 -> R712 -> N41 -> N214 -> V204 -> D161 -> V162 -> D93	20203
	21nt	C544 -> Q543 -> I565 -> L563 -> R790 -> Y788 -> S794 -> S750 -> C749 -> R759 -> R712 -> N41 -> N214 -> V204 -> D161 -> V162 -> D93	22071
hAgo2	apo	L522 -> Y529 -> K533 -> H816 -> A813 -> A810 -> A806 -> A803 -> I799 -> S763 -> P762 -> R714 -> N43 -> N216 -> V206 -> I159 -> A161 -> Y101	21349
	21nt	V547 -> Q545 -> I567 -> L565 -> R792 -> Y790 -> S796 -> S752 -> L750 -> F748 -> T741 -> S209 -> M213 -> M47	17556
hAgo3	apo	K534 -> R535 -> H817 -> A814 -> A811 -> D598 -> D670 -> V707 -> H767 -> D748 -> H743 -> A210 -> R208 -> V160 -> P159 -> V131	19727
	21nt	C547 -> Q546 -> I568 -> K567 -> R793 -> Y791 -> A802 -> I800 -> S764 -> R762 -> R715 -> N35	13974
hAgo4	apo	K525 -> T530 -> V848 -> A815 -> A812 -> A808 -> A805 -> I801 -> S765 -> R763 -> R716 -> N33	13878
	21nt	T518 -> P519 -> V820 -> H818 -> A815 -> A812 -> A808 -> A588 -> G671 -> Q710 -> H713 -> T715 -> N206 -> V196 -> D153 -> V154 -> D85	23156

[a] Ct: Commute time

Table S14. List of top 15 residues of PAZ-N shortest communication pathways weighted over the most representative structures of the top 3 clusters of hAgo apo conformations and 21nt miR-20a complexes. The top 15 residues conserved between apo structures and 21nt miR-20a complexes of each isoform are in bold characters. Table cells of different protein domains are colored using the same color code of **Figure 1**. The Beam domain residues are included in the N domain.

hAgo domain	hAgo1		hAgo2		hAgo3		hAgo4	
	apo	21nt	apo	21nt	apo	21nt	apo	21nt
N	V91	Y92	**F94**	V93	**G113**	V51	V49	G83
	E117	**H166**	**H168**	**F94**	**E114**	I53	I51	Y84
	G119	**S169**	**S171**	**H168**	G115	V66	V107	Y91
	E120	F179	P176	**S171**	**R119**	V109	L109	H158
L1	**H166**	**F180**	**F182**	T175	**F121**	L111	G111	S161
	**S169**	**W197**	E197	**F182**	V123	**G113**	E112	T165
	T173	F222	W199	V198	I125	**E114**	G113	F171
	**F180**	**K224**	F202	F200	**F183**	**R119**	Q116	W189
	E195	**V265**	I217	F224	E198	**F121**	F118	F214
	**W197**	C325	K226	**V267**	W200	**F183**	F192	V257
	**K224**	**V328**	**V267**	V281	K227	R278	I207	V271
PAZ	**V265**	**Y336**	E268	V330	**Y280**	**Y280**	K268	L318
	**V328**	L339	R280	Y338	**L297**	**L297**	Y269	Y328
	**Y336**	V345	L341	V347	**V331**	Y312	V320	V337
	I344	G347	I346	G349	I347	**V331**	G321	G339

Table S15 PAZ-N shortest communication pathways and commute times of the most representative structure of cluster 1 of hAgo apo conformations and 21-nt miR-20a complexes. The top 15 conserved residues are highlighted in red.

hAgo	sys	PAZ-N shortest communication pathway	Ct[a]
hAgo1	apo	T335 -> V328 -> V279 -> V265 -> I344 -> K224 -> E195 -> W197 -> F180 -> T173 -> S169 -> H166 -> V91 -> Y99	17360
	21nt	T335 -> V328 -> Y277 -> V265 -> V345 -> G347 -> F222 -> W197 -> F180 -> F179 -> S169 -> H166 -> Y92 -> Y99	19111
hAgo2	apo	T337 -> V330 -> R280 -> V267 -> I346 -> K226 -> E197 -> W199 -> F182 -> P176 -> S171 -> H168 -> F94 -> Y101	18143
	21nt	H336 -> Y338 -> V330 -> V281 -> V267 -> V347 -> G349 -> T175 -> S171 -> H168 -> V93 -> Y101	16504
hAgo3	apo	Q298 -> L297 -> Y312 -> A310 -> A290 -> P341 -> L329 -> V331 -> Y280 -> E114 -> G113 -> R119 -> F121 -> V123 -> I125 -> Y49	23888
	21nt	T338 -> V331 -> Y280 -> R278 -> E114 -> G113 -> R119 -> F121 -> I53 -> V51 -> Y49	17330
hAgo4	apo	T327 -> G321 -> G113 -> G111 -> Q116 -> F118 -> I51 -> V49 -> Y47	14858
	21nt	H326 -> Y328 -> L318 -> V271 -> V257 -> V337 -> G339 -> T165 -> S161 -> H158 -> G83 -> Y91	16604

[a] Ct: Commute time

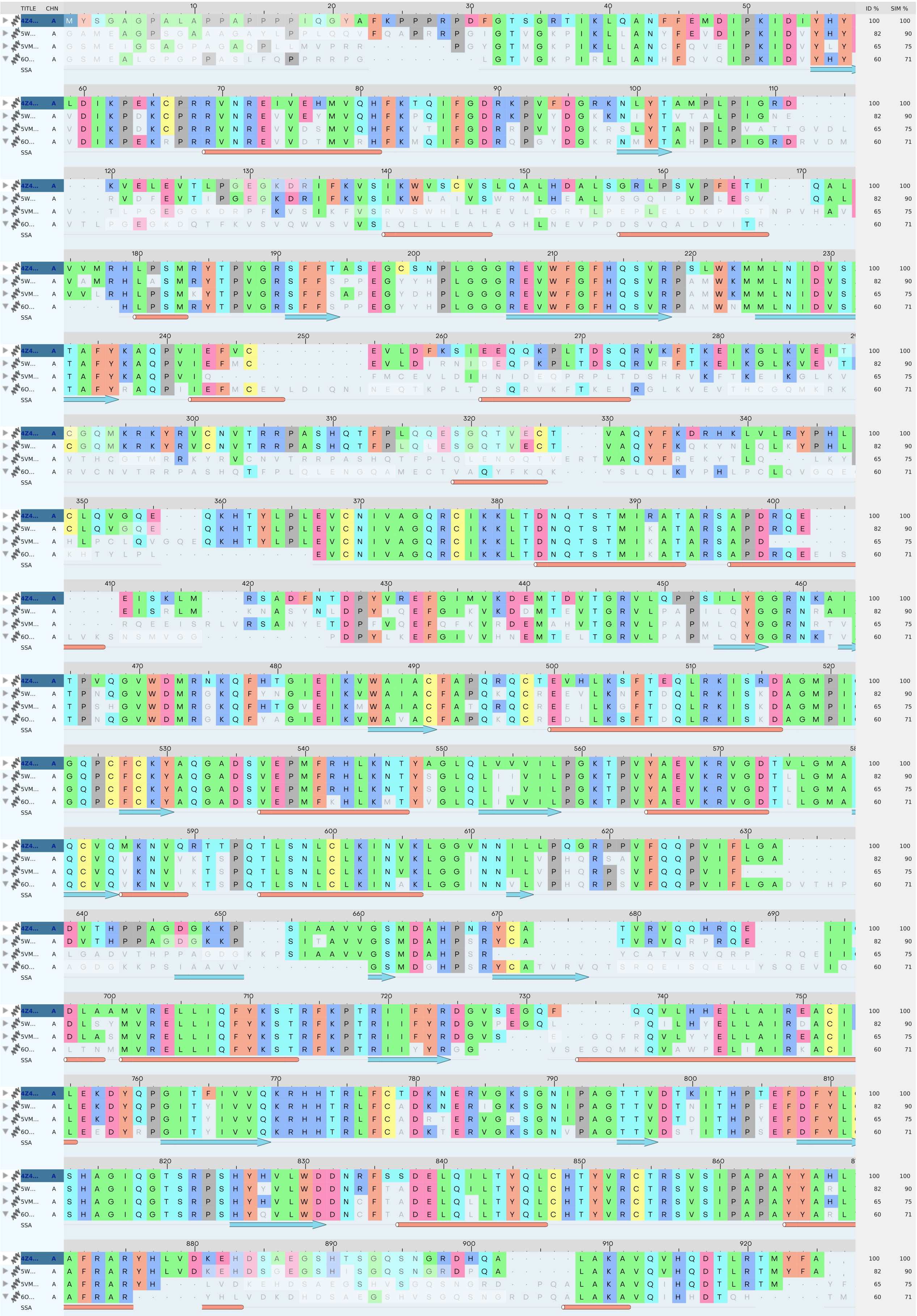

Figure S1. hAgo1-4 sequence alignment. hAgo2 (UniProt Q9UKV8) is the reference sequence, consensus is highlighted in sidechains based color codes, secondary structure elements are shown at the bottom.

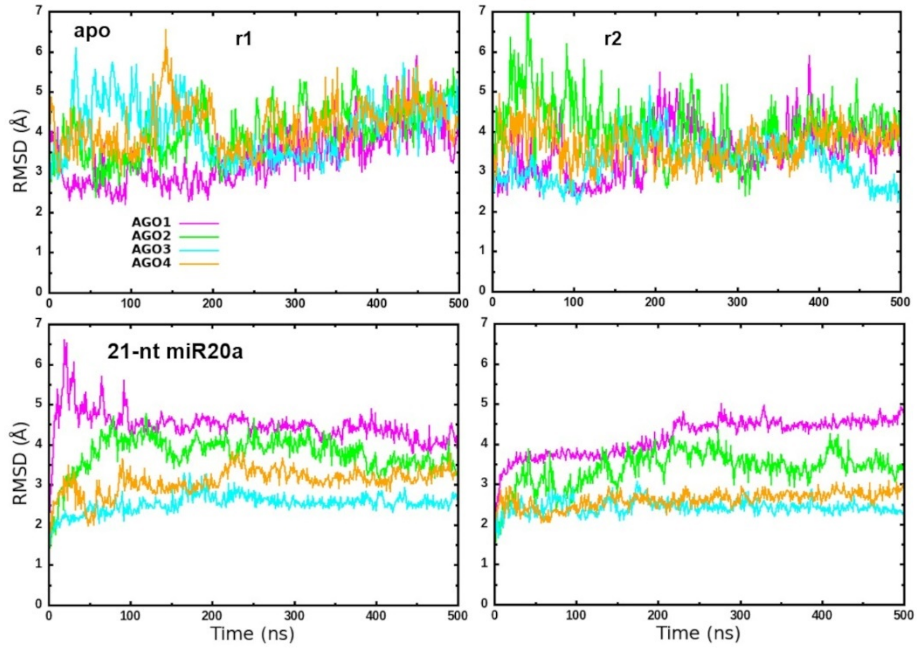

Figure S2. RMSD evolution. RMSD values calculated for Cα atoms *per* replica, namely r1 and r2, in the apo and RNA bound hAgo isoforms.

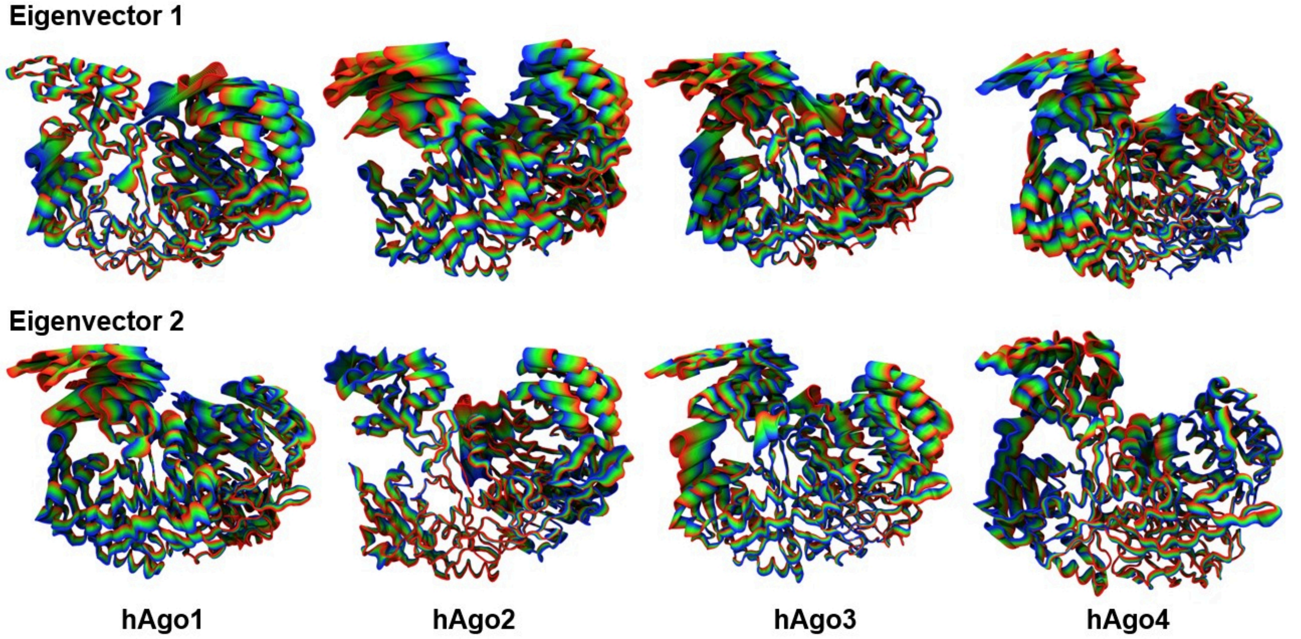

Figure S3. Principal Component Analysis (PCA) of hAgo isoforms apo conformations. Covariance matrix has been built on Cα atoms. The first two principal components (eigenvectors 1 and 2) are used for the projection on the average structures.

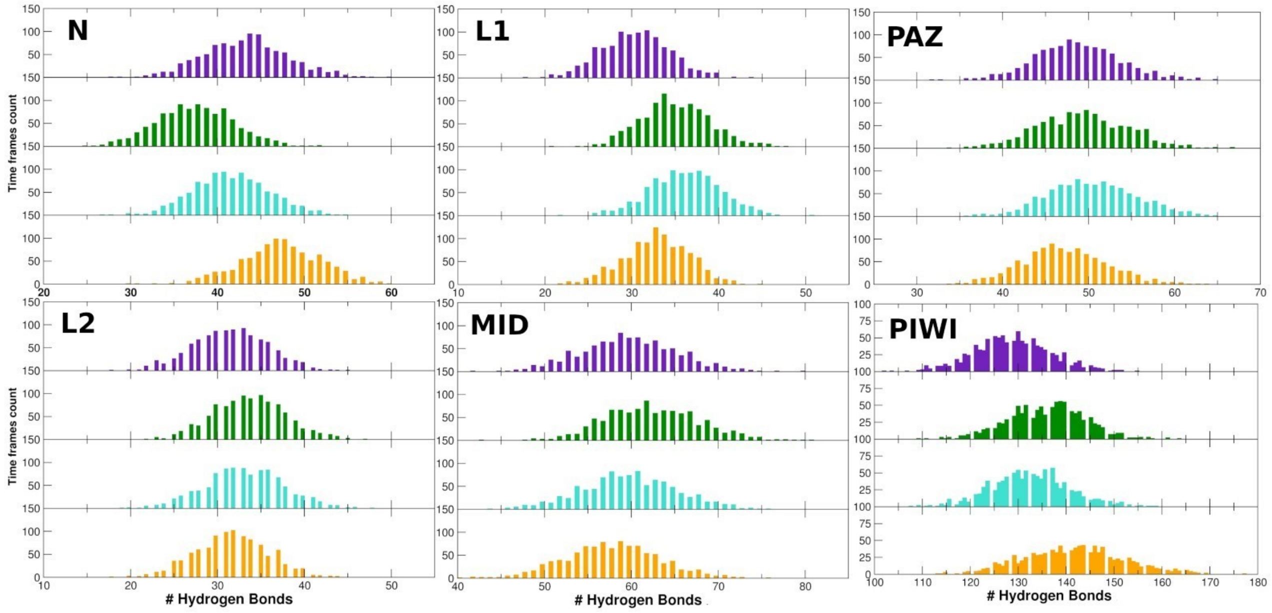

Figure S4. Intra-domain hydrogen bonds distribution *per* domain in hAgo isoforms apo conformations.

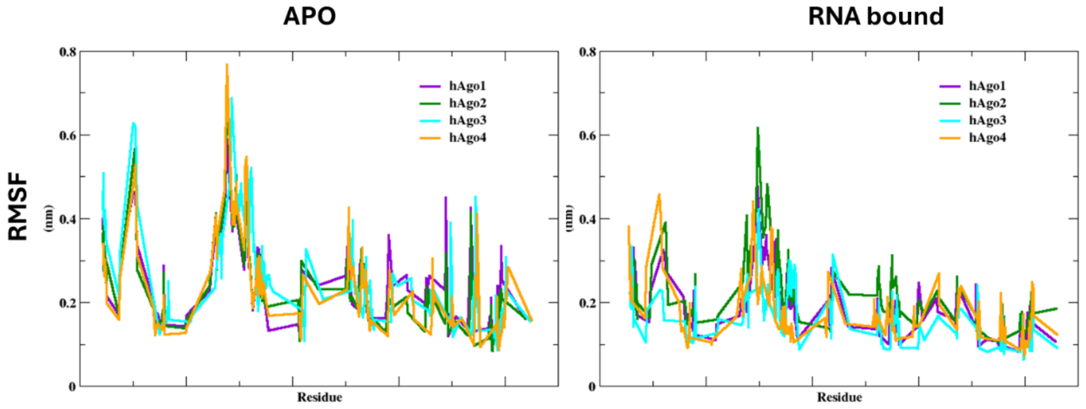

Figure S5. RMSFs of the RNA-binding cleft in the four hAgo isoforms in the apo state and in complex with 21-nt miR-20a.

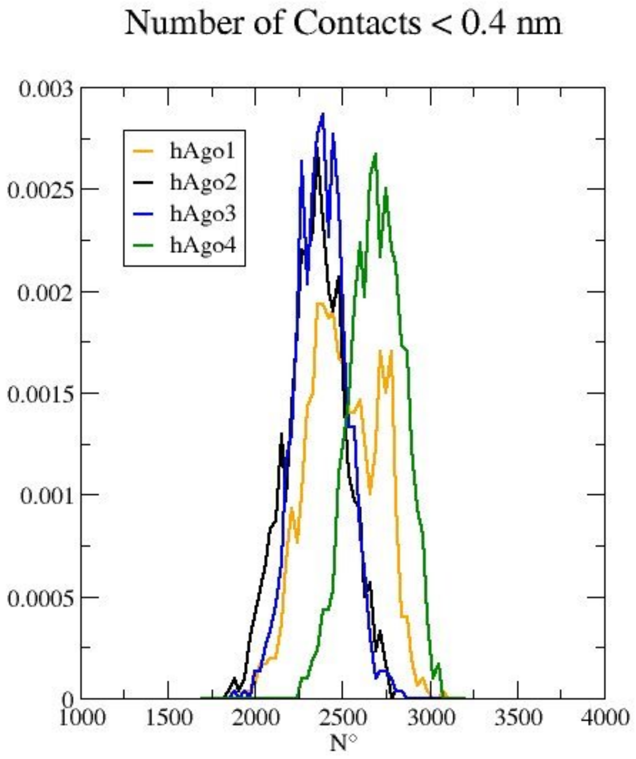

Figure S6. Number of contacts of RNA with the protein in the four hAgo 21-nt miR-20a complexes.

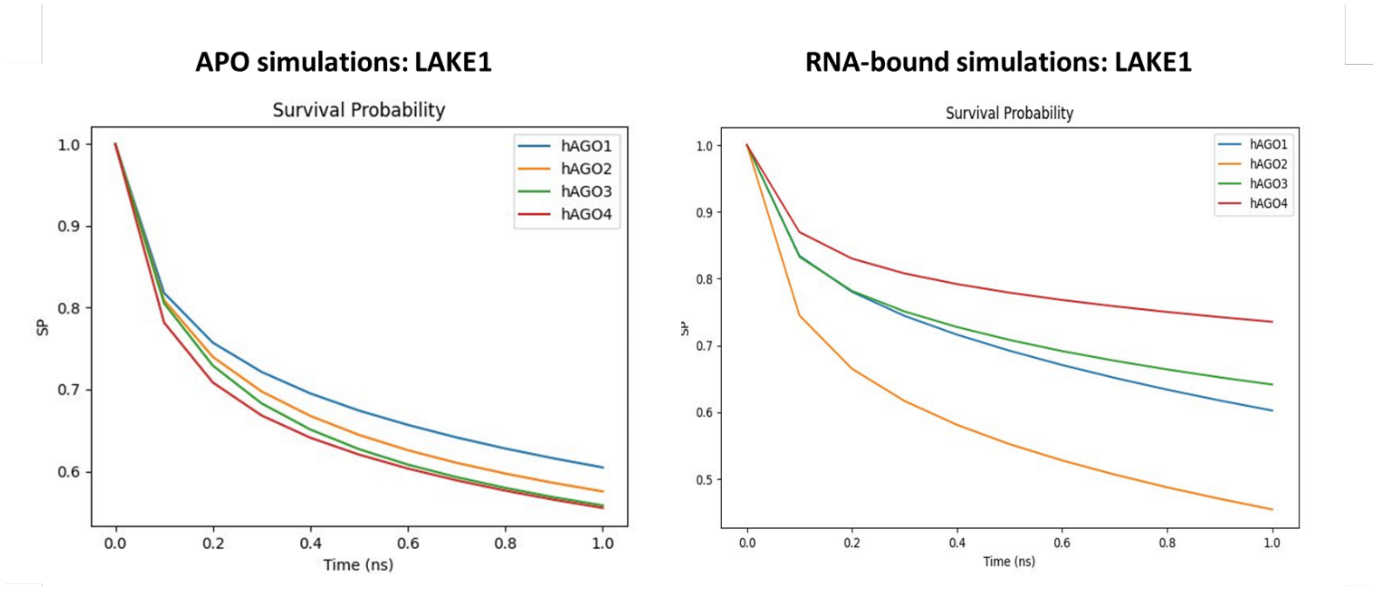

Figure S7. Survival probability (SP) of water molecules in LAKE1 pocket of the four hAgo isoforms in apo and RNA-bound states during MD simulations.

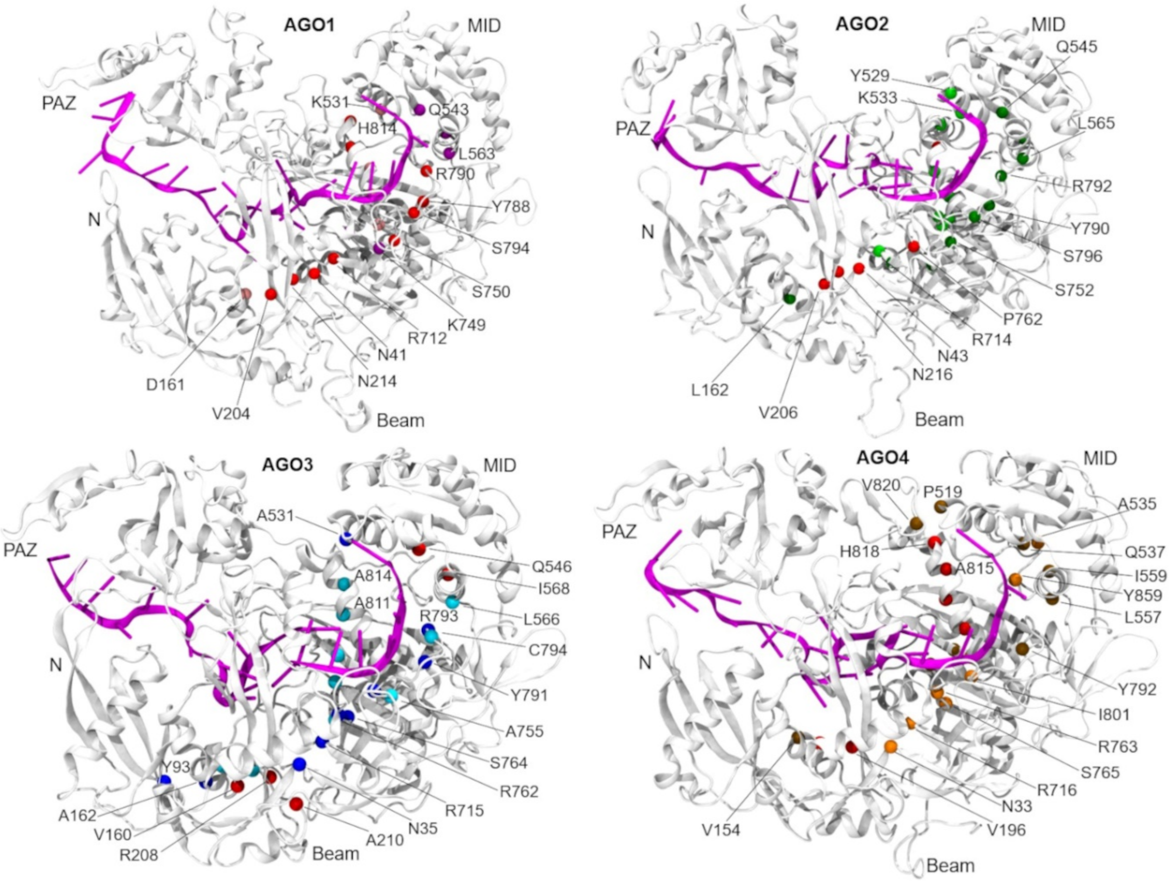

Figure S8. Top 15 residues of MID-N shortest communication pathways mapped on cluster 1 structures of human hAgo isoforms apo conformations and 21-nt miR-20a complexes. For clarity, only alpha carbons atoms of top 15 residues are shown in space filling representation. The top 15 residues of apo conformations are displayed with brighter colors (i.e., pink, light green, cyan and orange for hAgo1, hAgo2, hAgo3 and hAgo4, respectively). The top 15 residues of 21-nt miR-20a complexes are displayed with darker colors (i.e., purple, dark green, blue and brown for hAgo1, hAgo2, hAgo3 and hAgo4, respectively). The top 15 conserved residues between apo conformations and 21-nt miR-20a complexes are shown in red. Protein and RNA molecules are shown in white and magenta, respectively.

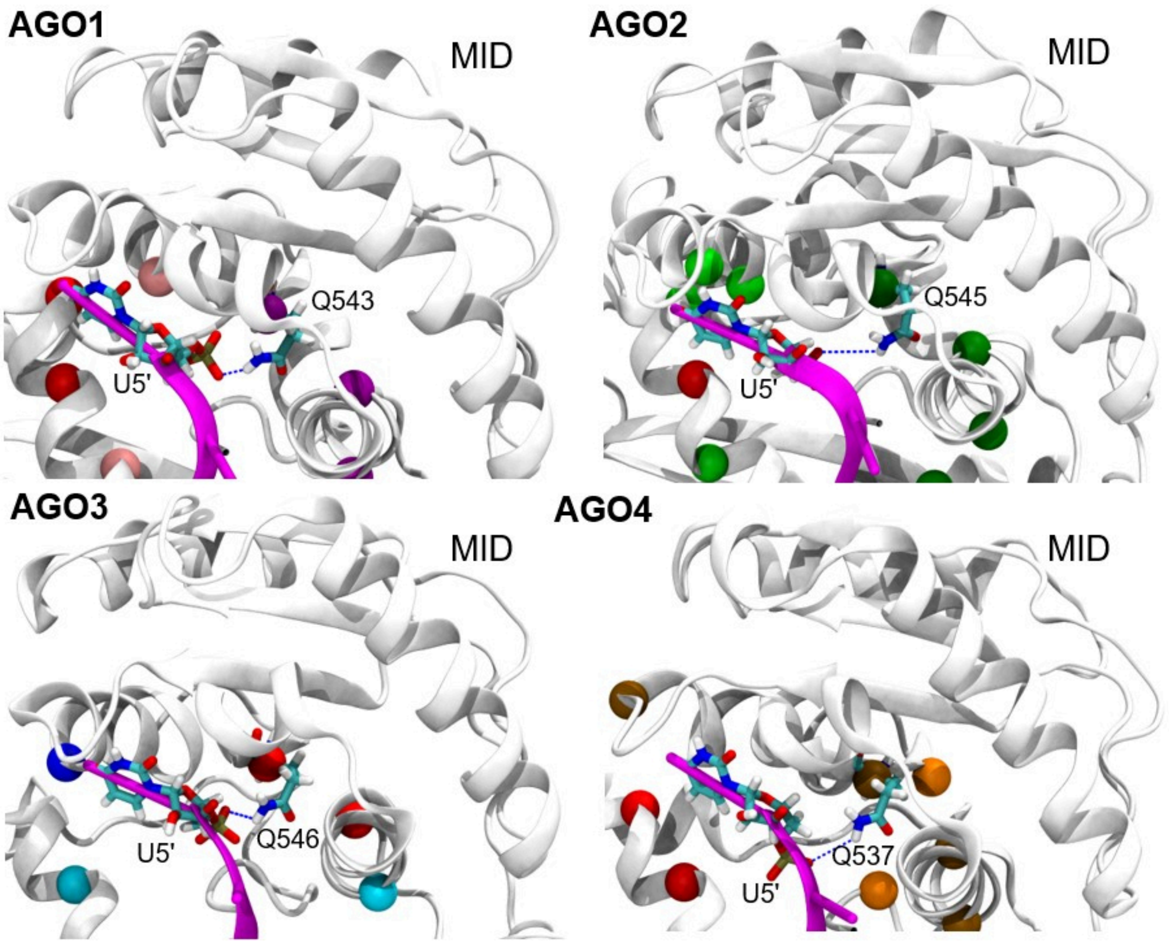

Figure S9. Hydrogen bond interactions of the conserved top 15 residue hAgo2 Q545 with miRNA 5' terminal phosphate (U5') in MID-Beam+N shortest communication pathways mapped on cluster 1 structures of human hAgo isoforms in complex with 21-nt miR-20a. For clarity, only a zoomed view of the MID domain from **Figure S8** is shown. The same color code of **Figure S8** is used to represent the top 15 residues, protein and RNA molecules.

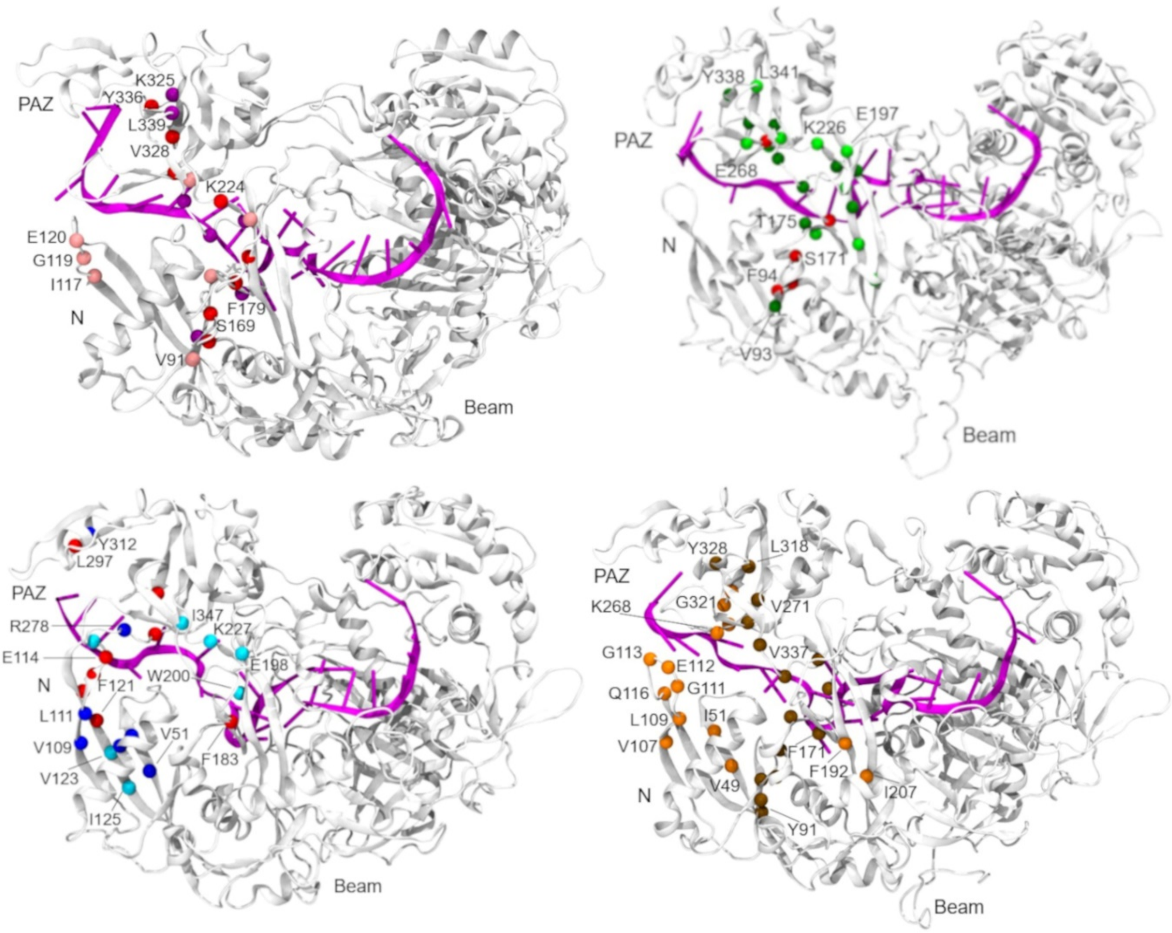

Figure S10. Top 15 residues of PAZ-N shortest communication pathways mapped on cluster 1 structures of human hAgo isoforms apo conformations and 21-nt miR-20a complexes. For clarity, only alpha carbons atoms of top 15 residues are shown in space filling representation. The same color code of **Figure S8** is used to represent the top 15 residues, protein and RNA molecules.

REFERENCES

[1] M.A. Larkin, G. Blackshields, N.P. Brown, R. Chenna, P.A. McGettigan, H. McWilliam, F. Valentin, I.M. Wallace, A. Wilm, R. Lopez, J.D. Thompson, T.J. Gibson, D.G. Higgins, Clustal W and Clustal X version 2.0, Bioinformatics 23 (2007) 2947–2948. https://doi.org/10.1093/bioinformatics/btm404.

[2] J.D. Thompson, D.G. Higgins, T.J. Gibson, CLUSTAL W: improving the sensitivity of progressive multiple sequence alignment through sequence weighting, position-specific gap penalties and weight matrix choice, Nucleic Acids Res. 22 (1994) 4673–4680. https://doi.org/10.1093/nar/22.22.4673.

[3] W. Humphrey, A. Dalke, K. Schulten, VMD: Visual molecular dynamics, J. Mol. Graph. 14 (1996) 33–38. https://doi.org/10.1016/0263-7855(96)00018-5.

[4] M.S. Park, R. Araya-Secchi, J.A. Brackbill, H.-D. Phan, A.C. Kehling, E.W. Abd El-Wahab, D.M. Dayeh, M. Sotomayor, K. Nakanishi, Multidomain Convergence of Argonaute during RISC Assembly Correlates with the Formation of Internal Water Clusters, Mol. Cell 75 (2019) 725-740.e6. https://doi.org/10.1016/j.molcel.2019.06.011.

[5] K. Nakanishi, Anatomy of four human Argonaute proteins, Nucleic Acids Res. 50 (2022) 6618–6638. https://doi.org/10.1093/nar/gkac519.

[6] P. Rice, I. Longden, A. Bleasby, EMBOSS: The European Molecular Biology Open Software Suite, Trends Genet. 16 (2000) 276–277. https://doi.org/10.1016/S0168-9525(00)02024-2.
